# Supplementary material for: The reliability of the angle of deviation measurement from the Photo-Hirschberg tests and Krimsky tests
Source: PLoS One. 2021 Dec 1;16(12):e0258744. doi: 10.1371/journal.pone.0258744 (PMC8635364; doi:10.1371/journal.pone.0258744)
Supplement: S1 File — (PDF) [file pone.0258744.s001.pdf]

EC: 56-167-02-4-2

คณะแพทยศาสตร์ มหาวิทยาลัยสงขลานครินทร์
ตำบลคอหงส์ อำเภอหาดใหญ่
จังหวัดสงขลา 90110

หนังสือรับรองนี้ให้ไว้เพื่อแสดงว่า

โครงการวิจัยเรื่อง : การเปรียบเทียบค่ามุมเขจากการใช้ภาพนิ่งวัดแสงสะท้อนผิวกระจกตา กับค่ามุมเขจากการวัดปริซึม ในผู้ป่วยตาเข (The Comparison an Angle of Deviation from Photographs with Alternate Prism Cover Test in Strabismic Patients)

หัวหน้าโครงการ : นายแพทย์อรรคพล ตั้งสัตยาธิษฐาน

ภาควิชา/คณะ : ภาควิชาจักษุวิทยา คณะแพทยศาสตร์ มหาวิทยาลัยสงขลานครินทร์

ได้ผ่านการพิจารณาและได้รับความเห็นชอบจากคณะกรรมการอนุกรรมการจริยธรรมด้านวิจัยเกี่ยวกับบริบาลผู้ป่วย สิ่งส่งตรวจ และสังคมศาสตร์ ของคณะแพทยศาสตร์ มหาวิทยาลัยสงขลานครินทร์ แล้ว

ให้ไว้ ณ วันที่ 5 เมษายน 2556

....ประธานอนุกรรมการ
(รองศาสตราจารย์นายแพทย์บุญสิน ตั้งตระกูลวนิช)
รองคณบดีฝ่ายวิจัย

FACULTY OF MEDICINE
PRINCE OF SONGKLA UNIVERSITY

EC: 56-167-02-4-2

Documentary Proof of Ethical Clearance

The Ethics Committee, Faculty of Medicine, Prince of Songkla University

The Project Entitled	:	The Comparison an Angle of Deviation from Photographs with Alternate Prism Cover Test in Strabismic Patients
Principal Investigator	:	Akkapol Tungsattayathithan, M.D.
Name of Department	:	Department of Ophthalmology, Faculty of Medicine, Prince of Songkla University

has been reviewed and approved by The Ethics Committee, Faculty of Medicine, Prince of Songkla University.

Date of Approval : April 5, 2013

....

(Assoc.Prof. Boonsin Tangtrakulwanich)

Associate Dean for Research Affairs
