# Supplementary material for: The reliability of the angle of deviation measurement from the Photo-Hirschberg tests and Krimsky tests
Source: PLoS One. 2021 Dec 1;16(12):e0258744. doi: 10.1371/journal.pone.0258744 (PMC8635364; doi:10.1371/journal.pone.0258744)
Supplement: S2 File — (PDF) [file pone.0258744.s002.pdf]

เฉพาะเจ้าหน้าที่ฝ่ายวิจัย

รหัสโครงการวิจัยฯ	☐☐-☐☐☐-☐☐-☐☐-☐<br>ป ป ล ล ล ภ ภ บ บ จธ

ป = ปีงบประมาณ	ล = ลำดับเรียงก่อนหลัง	ภ = ภาควิชา/หน่วยงาน	บ = ประเภทบุคลากร	จธ = จริยธรรม

ส่งเรื่องจากหน่วยงาน	☐☐-☐☐☐☐<br>ว ว ด ด ป ป	ฝ่ายวิจัยได้รับ	☐☐-☐☐☐☐<br>ว ว ด ด ป ป
ส่ง reviewer	☐☐-☐☐☐☐	รับจาก reviewer	☐☐-☐☐-☐☐
ลงนามใบรับรอง	☐☐-☐☐☐☐<br>ว ว ด ด ป ป	หน่วยงานได้รับหนังสือรับรอง	☐☐-☐☐-☐☐<br>ว ว ด ด ป ป

ตรวจเลือกข้อหนึ่งข้อใด (วงเงินมากกว่า 100,000 บาท พิจารณาใน กก กลั่นกรอง

วงเงินที่ได้	[ ]		บาท	→	[ ]	ผ่านฝ่ายวิจัย	☐☐-☐☐-☐☐
วงเงินที่ได้	[ ]		บาท	→	[ ]	ผ่าน กก. กลั่นกรอง	☐☐-☐☐-☐☐

รับเงินจากงานคลัง	☐☐-☐☐☐☐	ใช้จริงหลังเสร็จสิ้น		บาท

แบบข้อเสนอโครงการวิจัย <u>บริบาล</u> พร้อมสิ่งที่ต้องเสนอฯ

คณะอนุกรรมการพิจารณาจริยธรรมด้านวิจัยเกี่ยวกับการบริบาลผู้ป่วย สิ่งส่งตรวจ และสังคมศาสตร์การแพทย์

คณะกรรมการกลั่นกรองและพิจารณาเงินอุดหนุนการวิจัย - พิจารณาทุนอุดหนุนฯ (ถ้าเกี่ยวข้อง)

1 **ชื่อโครงการวิจัย** (ภาษาไทย)

การเปรียบเทียบค่ามุมเขจากการใช้ภาพนิ่งวัดแสงสะท้อนผิวกระจกตา กับค่ามุมเขจากการวัดปริซึม ในผู้ป่วยตาเข

Title (ภาษาอังกฤษ)

The comparison an angle of deviation from photographs with alternate prism cover test in strabismic patients

2 **คำสำคัญ 2-5 คำ** (ภาษาไทย)

แสงสะท้อนผิวกระจกตา, ปริซึม, ตาเข, มุมเข, ภาพถ่าย

Keywords (ภาษาอังกฤษ)

Hirschberg test, alternate prism cover test, horizontal strabismus, angle of deviation, photographs

3 **ลักษณะข้อเสนอโครงการวิจัยฯ** เพื่อการรับรองด้านจริยธรรมแบบรวดเร็ว (สามารถตรวจเลือกได้มากกว่าหนึ่งข้อ)

3.1. โครงการวิจัยฯที่มี **ความเสี่ยงต่ำ** (low risk) และเป็นการ **บริบาล** ที่ **ไม่อยู่** ในแนวทางการดูแลรักษาของประเทศไทย พร้อมกับรวบรวมข้อมูลเชิงวิจัย ดังกรณีต่อไปนี้

☐ 3.1.1. การศึกษา **ยาที่ขึ้นทะเบียน** แล้ว แต่ศึกษา **ขนาดยา/วิธีการ/กลุ่มประชากรใหม่** ที่ไม่ได้ระบุอยู่ในการขึ้นทะเบียนนั้น โดย **มี** ผลการศึกษาในประเทศอื่นมาก่อน

☑ 3.1.2. การศึกษา **เทคนิคการผ่าตัด/วิธีการรักษา/อุปกรณ์ใหม่** โดย **มี** ผลการศึกษาในประเทศอื่นมาก่อน

สิ่งที่ต้องเสนอพร้อมข้อเสนอโครงการวิจัยฯ (โปรดระบุ)

☒ **ผลการศึกษาในประเทศอื่นที่เกี่ยวข้อง/คล้ายคลึง (บทคัดย่อ 1 ถึง 2 เรื่อง)**

☐ ได้รับการรับรองจากองค์กรวิชาชีพ/วิชาการที่เกี่ยวข้อง

☒ **มี/จัดให้มีกระบวนการลงนามยินยอมอย่างเต็มที่ (3.1.1. และ 3.1.2)**

☒ **ใบยินยอมที่มีรายละเอียด ตามแบบเอกสารแนะนำผู้ป่วย/ลงนามยินยอม (3.1.1. และ 3.1.2.)** โดยหลีกเลี่ยงการใช้ศัพท์แพทย์/ภาษาอังกฤษให้มากที่สุด

☑ มีแบบบันทึกข้อมูล

☒ รับรู้รับทราบข้อ 13 เกี่ยวกับการรายงานเหตุการณ์ไม่พึงประสงค์ที่ร้ายแรง

☐ มีเกณฑ์ให้ยุติการศึกษาเป็นการเฉพาะราย (เปลี่ยนเป็นการรักษาอื่นด้วยเงื่อนไขใด)

☐ แบบฟอร์ม จธ 06 (2554) เก็บตัวอย่างชีวภาพตรวจ ณ ต่างประเทศ (ถ้าเกี่ยวข้อง)

☐ แบบฟอร์ม จธ 07 (2554) เก็บตัวอย่างชีวภาพเพื่อศึกษาวิจัยในอนาคต (ถ้าเกี่ยวข้อง)

หมายเหตุ แบบฟอร์ม จธ 06 (2554), จธ 07 (2554) หลังจากผู้เข้าร่วมการศึกษาลงนามยินยอม ให้หัวหน้าโครงการวิจัยฯส่งตัวจริง/สำเนาแก่คณะกรรมการจริยธรรมด้านวิจัย 1 ชุด เป็นรายเดือน ผ่านฝ่ายวิจัย

กระบวนการลงนามยินยอมอย่างเต็มที่ (3.1.1. และ 3.1.2)

ยึดหลัก **“ผู้ใหญ่ทุกคนที่มีอายุครบบริบูรณ์มีสิทธิในการกำหนดว่า อะไรควรทำเกี่ยวกับร่างกายของเขา หรือ ผู้เยาว์ของเขา”** หรือ หลัก **การปฏิเสธการยินยอม** ผู้ป่วยมีทั้งสิทธิที่จะกำหนดว่า ควรทำ/ไม่ควรทำอะไรกับร่างกายของเขา

ยึดหลัก **มาตรฐานผู้ป่วยอันเป็นเหตุเป็นผล** (reasonable patient standard) ภายใต้มาตรฐานนี้ หัวหน้าโครงการวิจัย/ผู้ร่วมวิจัยถูกร้องขอ (require) ให้บอกผู้ป่วยทุกสิ่งที่รองรับอย่างเป็นเหตุเป็นผล อันเกี่ยวข้องกับการตัดสินใจยอมรับการรักษา/เลือกรักษา ตามกฎทั่วไป วิธีการปฏิบัติในกรณีโครงการวิจัยฯ เป็นวิธีการที่มีทางเลือกรักษาหลายอย่าง มีรายละเอียดที่ต้องเปิดเผยมากกว่า

แนวทาง

1. **ค่าใช้จ่ายนอกเหนือจากสิทธิประโยชน์ในการรักษาพยาบาลของผู้เข้าร่วมการศึกษา โครงการวิจัยฯเป็นผู้รับผิดชอบ** ด้วยเงินอุดหนุนการวิจัย จากกองทุนวิจัย คณะแพทยศาสตร์ **หรือ** แหล่งทุนอื่น
2. อธิบายต่อผู้ป่วยเกี่ยวกับการวินิจฉัยและธรรมชาติของโรค/ภาวะ/การบาดเจ็บ ที่ต้องได้รับการปฏิบัติการทางการแพทย์
3. อธิบายผู้ป่วยเกี่ยวกับธรรมชาติและวัตถุประสงค์ของการรักษา/เลือกรักษา
4. อธิบายอย่างครบถ้วนเกี่ยวกับความเสี่ยงอันเป็นที่ทราบกันดี และผลที่ติดตามมาเกี่ยวเนื่องกับการ

รักษาที่หัวหน้าโครงการวิจัยฯ/นักวิจัยแนะนำ

5. อธิบายอย่างครบถ้วนถึงทางเลือกรักษาที่ดำเนินการได้ รวมถึงทางเลือกที่จะไม่รักษาและอธิบายถึงความเสี่ยงและภาวะแทรกซ้อนเกี่ยวกับทางเลือกรักษา
6. อธิบายด้วยคำศัพท์อันเป็นที่เข้าใจและประสบความสำเร็จในการทำเข้าใจร่วมกัน
7. ยอมให้ผู้ป่วยมีเวลาพอสมควรในการไตร่ตรองข้อมูลที่ได้รับ และกระตุ้นให้ตั้งคำถามเพื่อทำให้มั่นใจว่าผู้ป่วยเข้าใจข้อมูลที่ได้รับ
8. ควรได้รับการลงนามรับรองโดยผู้ป่วยเพื่อบ่งชี้ว่า ได้รับทราบและเข้าใจข้อมูลที่ได้รับ

เอกสารแนะนำผู้ป่วย/ลงนามยินยอม (3.1.1. และ 3.1.2.)

ส่วนข้อมูลทั่วไป

- วันเดือนปี
- ชื่อหัวหน้าโครงการวิจัยฯ
- ชื่อโครงการวิจัย
- รหัสโครงการวิจัยฯ (ลงภายหลัง)
- แหล่งทุนวิจัย
- ชื่อผู้ยินยอม ที่อยู่

ส่วนโครงการวิจัยฯ

- โรค/ภาวะ/การบาดเจ็บของผู้เข้าร่วมโครงการวิจัยฯ
- แนวทางการดูแลรักษาของประเทศไทย (ทางเลือกเดียว/หลายทางเลือก) และภาวะแทรกซ้อนสำคัญ
- การรักษา/ผ่าตัด/ใช้อุปกรณ์ (เลือก/สุ่มเลือกสู่โครงการวิจัยฯ) โดย **ไม่มี** ผลการศึกษาในประเทศไทยมาก่อน แต่ **มี** ผลการศึกษาในต่างประเทศมาก่อน มีภาวะแทรกซ้อน/ข้อได้เปรียบ/เสียเปรียบอะไรบ้าง เมื่อเปรียบเทียบกับแนวทางการรักษา/ผ่าตัด/ใช้อุปกรณ์ในปัจจุบัน
- ในกรณีเป็นการสุ่มอิสระเพื่อเปรียบเทียบผลการรักษา ให้อธิบายความแตกต่างระหว่างกลุ่ม
- **ค่าใช้จ่ายนอกเหนือจากสิทธิประโยชน์ในการรักษาพยาบาลของผู้เข้าร่วมการศึกษา**

ส่วนการยินยอม

- หากต้องการเก็บตัวอย่างทางชีวภาพของข้าพเจ้า เพื่อส่งตรวจ ณ ห้องปฏิบัติการ ณ ต่างประเทศ เพื่อการศึกษาวิจัยในอนาคต จะเป็นเพียงการตรวจ/เก็บรักษาไว้เพื่อตรวจทางอณูพันธุกรรมและวิทยาศาสตร์พื้นฐาน ภายใต้การยินยอมและลงนามในใบยินยอมของข้าพเจ้า ตามแบบฟอร์ม จธ 06 (2554) เก็บตัวอย่างชีวภาพตรวจ ณ ต่างประเทศ หรือ แบบฟอร์ม จธ 07 (2554) เก็บตัวอย่างชีวภาพเพื่อศึกษาวิจัยในอนาคต
- ในกรณีที่ข้าพเจ้ามีข้อสงสัยเกี่ยวกับโครงการวิจัยฯ ข้าพเจ้ามีสิทธิซักถามหัวหน้าโครงการวิจัย/นักวิจัยได้ในระหว่างการดูแลรักษา เมื่อไรก็ตาม การกระทำและคำชี้แจงของหัวหน้าโครงการวิจัย/นักวิจัยยังไม่เป็นที่กระจ่างชัด ข้าพเจ้าสามารถแจ้งต่อผู้รับผิดชอบในระดับที่สูงกว่า โดยแจ้งต่อประธานคณะกรรมการจริยธรรมด้านวิจัยเกี่ยวกับการทดลองต่อผู้เข้าร่วมการศึกษา (คณบดี คณะแพทยศาสตร์ โทร.074-451-100) หรือรองประธานฯ (รองคณบดีฝ่ายวิจัย โทร 074-451-149)
- ข้าพเจ้ารับทราบว่า ข้าพเจ้าสามารถติดต่อหัวหน้าโครงการวิจัยฯได้ทุกเมื่อที่โทรศัพท์มือถือ .... หรือนักวิจัย/ผู้ช่วยวิจัยชื่อ .... ได้ที่ (ชื่อหน่วยงาน) .... มือถือ ....
- ข้าพเจ้าได้รับทราบว่า ข้อมูลเฉพาะบุคคล โรค/ภาวะ/การบาดเจ็บ การดำเนินโรค ข้อมูลโครงการวิจัยฯของข้าพเจ้า ได้รับการเก็บรักษาเป็นความลับ โดยมีการปกปิดชื่อ/ข้อมูลที่สามารถอ้างอิงถึงตัวบุคคลได้

และไม่มีผู้ใดเข้าถึงข้อมูลเหล่านี้ได้ นอกจากทีมนักวิจัย เว้นแต่จะได้รับความยินยอมจากข้าพเจ้า ทั้งนี้ในระหว่าง/สิ้นสุดโครงการวิจัยฯ หากปรากฏข้อมูลโรค/ภาวะ/การบาดเจ็บ การดำเนินโรค อันเป็นประโยชน์ต่อสุขภาพ เป็นหน้าที่ของหัวหน้าโครงการวิจัยฯต้องแจ้งให้ข้าพเจ้ารับรู้รับทราบ

- ในกรณีมีทีมผู้ตรวจสอบข้อมูลโครงการวิจัยฯจากแหล่งทุนวิจัย หัวหน้าโครงการวิจัยฯ/นักวิจัยต้องให้ข้าพเจ้าได้รับทราบล่วงหน้าก่อนเข้าสู่โครงการวิจัยฯ และหัวหน้าโครงการวิจัยฯ/นักวิจัยต้องจัด/จัดให้มีระบบการรักษาความลับของข้าพเจ้า ในกรณีที่ข้อมูลส่วนตัวของข้าพเจ้าถูกเปิดเผยต่อสาธารณะ หรือเป็นข้อมูลอันเป็นมูลเหตุให้สามารถระบุถึงตัวข้าพเจ้าได้ หัวหน้าโครงการวิจัยฯ/นักวิจัยต้องเป็นผู้รับผิดชอบความเสียหายต่อชื่อเสียงของข้าพเจ้า
- หากข้าพเจ้าไม่ประสงค์เข้าร่วมโครงการวิจัยอีกต่อไป ข้าพเจ้ามีสิทธิปฏิเสธการวิจัยด้วยการแจ้งต่อหัวหน้าโครงการวิจัย/นักวิจัย **หรือ** คณะกรรมการจริยธรรมด้านวิจัย เพื่อดำเนินการตามความประสงค์ของข้าพเจ้า และการปฏิเสธดังกล่าวก็จะไม่เสียสิทธิในการดูแลรักษาในโรงพยาบาลสงขลานครินทร์
- ข้าพเจ้าได้อ่านและเข้าใจเกี่ยวกับโครงการวิจัยฯตามคำอธิบายข้างต้นแล้ว ข้าพเจ้ายินยอมเข้าสู่โครงการวิจัยฯด้วยความสมัครใจ
- ผู้บรรลุนิติภาวะลงนามให้การยินยอม
- ผู้เยาว์อายุตั้งแต่ 15 ปีขึ้นไป แต่ไม่ถึง 20 ปี ผู้ปกครองลงนามให้การยินยอม ผู้เยาว์ลงนามรับทราบ
- ผู้เยาว์อายุน้อยกว่า 15 ปี ผู้ปกครองลงนามให้การยินยอม
- ผู้ขอการยินยอมลงนาม
- พยานลงนาม 2 คน

เอกสารแนะนำผู้ป่วย/ลงนามยินยอม (3.1.1. และ 3.1.2.) สำหรับการตรวจสอบย้อนหลัง (post audit)

ตามมติคณะกรรมการจริยธรรมด้านวิจัยฯ คราวประชุมครั้งที่ 10/2553 วันจันทร์ที่ 18 ตุลาคม 2553 และครั้งที่ 1/2554 ให้ถือปฏิบัติ ดังนี้

1. แบบฟอร์มเก็บตัวอย่างชีวภาพเพื่อศึกษาวิจัยในอนาคต ให้หัวหน้าโครงการวิจัยฯจัดทำเป็น 3 ชุด **ชุดแรก** ให้ผู้เข้าร่วมการศึกษา/ญาติ **ชุดที่สอง** หัวหน้าโครงการวิจัยฯเก็บรักษาไว้ และ **ชุดที่สาม** ส่งฝ่ายวิจัย
2. ฝ่ายวิจัยเป็นผู้เก็บรักษาทั้งแบบเอกสารและไฟล์เอกสาร พร้อมต่อการเรียกตรวจของคณะกรรมการฯ

3.2. โครงการวิจัยฯที่มี **ความเสี่ยงต่ำ** (low risk) และเป็นการ **บริบาล** ที่ **อยู่** ในแนวทางการดูแลรักษาของประเทศไทย พร้อมกับรวบรวมข้อมูลเชิงวิจัย ดังกรณีต่อไปนี้

☐ 3.2.1. การศึกษายาหลังจากยาได้รับการขึ้นทะเบียน ด้วยข้อบ่งชี้ที่ได้กำหนดไว้ในการขึ้นทะเบียน

☐ 3.2.2. การศึกษาการผ่าตัด/วิธีการรักษาตามแบบแผนที่เป็นที่ยอมรับและใช้อยู่ในปัจจุบัน

สิ่งที่ต้องเสนอพร้อมข้อเสนอโครงการวิจัยฯ (โปรดระบุ)

☒ **มี/จัดให้มีกระบวนยินยอมด้วยวาจาอย่างเต็มที่ (3.2.1. และ 3.2.2.)**

☒ มี/จัดให้มีการลงใบยินยอมของโรงพยาบาล [อยู่โรงพยาบาล หรือ การวิธีปฏิบัติที่รุกล้ำ (invasive procedure)]

☒ มีแบบบันทึกข้อมูล

☐ รับทราบข้อ 13 เกี่ยวกับการรายงานเหตุการณ์ไม่พึงประสงค์ที่ร้ายแรง

☐ แบบฟอร์ม จธ 06 (2554) เก็บตัวอย่างชีวภาพตรวจ ณ ต่างประเทศ (ถ้าเกี่ยวข้อง)

☐ แบบฟอร์ม จธ 07 (2554) เก็บตัวอย่างชีวภาพเพื่อศึกษาวิจัยในอนาคต (ถ้าเกี่ยวข้อง)

หมายเหตุ แบบฟอร์ม จธ 06 (2554), จธ 07 (2554) หลังจากผู้เข้าร่วมการศึกษาลงนามยินยอม ให้หัวหน้าโครงการวิจัยฯส่งตัวจริง/สำเนาแก่คณะกรรมการจริยธรรมด้านวิจัย 1 ชุด เป็นรายเดือน ผ่านฝ่ายวิจัย

กระบวนยินยอมด้วยวาจาอย่างเต็มที่ (3.2.1. และ 3.2.2)

ยึดหลัก **"ผู้ใหญ่ทุกคนที่มีอายุครบบริบูรณ์มีสิทธิในการกำหนดว่า อะไรควรทำเกี่ยวกับร่างกายของเขา หรือ ผู้เยาว์ของเขา"** หรือ หลัก **การปฏิเสธการยินยอม** ผู้ป่วยมีทั้งสิทธิที่จะกำหนดว่า ควรทำ/ไม่ควรทำอะไรกับร่างกายของเขา

ยึดหลัก **มาตรฐานผู้ป่วยอันเป็นเหตุเป็นผล** (reasonable patient standard) ภายใต้มาตรฐานนี้หัวหน้าโครงการวิจัยฯ/นักวิจัยถูกร้องขอ (require) ให้บอกผู้ป่วยทุกสิ่งที่รองรับอย่างเป็นเหตุเป็นผล อันเกี่ยวข้องกับการตัดสินใจยอมรับการรักษา/เลือกรักษา ตามกฎทั่วไป วิธีการปฏิบัติในกรณีโครงการวิจัยฯ เป็นวิธีการที่มีทางเลือกรักษาหลายอย่าง มีรายละเอียดที่ต้องเปิดเผยมากกว่า

แนวทาง

1. **ค่าใช้จ่ายนอกเหนือจากสิทธิประโยชน์ในการรักษาพยาบาลของผู้เข้าร่วมการศึกษา โครงการวิจัยฯเป็นผู้รับผิดชอบ** ด้วยเงินอุดหนุนการวิจัย จากกองทุนวิจัย คณะแพทยศาสตร์ **หรือ** แหล่งทุนอื่น
2. อธิบายต่อผู้ป่วยเกี่ยวกับการวินิจฉัยและธรรมชาติของโรค/ภาวะ/การบาดเจ็บ ที่ต้องได้รับการปฏิบัติการทางการแพทย์
3. อธิบายผู้ป่วยเกี่ยวกับธรรมชาติและวัตถุประสงค์ของการรักษา/เลือกรักษา
4. อธิบายอย่างครบถ้วนเกี่ยวกับความเสี่ยงอันเป็นที่ทราบกันดี และผลที่ติดตามมาเกี่ยวเนื่องกับการรักษาที่หัวหน้าโครงการวิจัยฯ/นักวิจัยแนะนำ
5. อธิบายอย่างครบถ้วนถึงทางเลือกรักษาที่ดำเนินการได้ รวมถึงทางเลือกที่จะไม่รักษาและอธิบายถึงความเสี่ยงและภาวะแทรกซ้อนเกี่ยวกับทางเลือกรักษา
6. อธิบายด้วยคำศัพท์อันเป็นที่เข้าใจและประสบความสำเร็จในการทำเข้าใจร่วมกัน
7. ยอมให้ผู้ป่วยมีเวลาพอสมควรในการไตร่ตรองข้อมูลที่ได้รับ และกระตุ้นให้ตั้งคำถามเพื่อทำให้มั่นใจว่าผู้ป่วยเข้าใจข้อมูลที่ได้รับ
8. ควรได้รับการลงนามรับรองโดยผู้ป่วยเพื่อบ่งชี้ว่า ได้รับทราบและเข้าใจข้อมูลที่ได้รับ

การพิจารณารับรอง

ในกรณีที่อนุกรรมการผู้ทบทวนสรุป **"เห็นชอบ"** ให้ประธานคณะอนุกรรมการจริยธรรมฯออกเอกสารรับรองการวิจัยสุขภาพ (certificate of health research approval) ให้แก่หัวหน้าโครงการวิจัยฯ ส่วนโครงการวิจัยฯที่ผ่านการรับรองให้ฝ่ายวิจัยสรุปในแบบฟอร์ม รหัส xx-04-03-02 (จธ 02) แจ้งให้คณะอนุกรรมการฯทราบเป็นรายเดือน ในกรณีที่มีข้อสังเกต/ข้อพิจารณามากพอต่อการพิจารณา ให้ประธานฯนำเข้าสู่การพิจารณาของกรรมการจริยธรรมเกี่ยวกับการทดลองต่อผู้เข้าร่วมการศึกษาตัดสิน

ในกรณีที่อนุกรรมการผู้ทบทวนสรุปพิจารณาเห็นว่า มีประเด็นด้านจริยธรรมที่ไม่กระจ่างใน 4 ประเด็นหลักได้แก่ **ความปลอดภัยของผู้เข้าร่วมการศึกษา การคุ้มครองสิทธิของผู้เข้าร่วมการศึกษา ความเป็นอยู่ที่ดีของผู้เข้าร่วมการศึกษา และการเคารพศักดิ์ศรีความเป็นมนุษย์** อนุกรรมการผู้ทบทวนสามารถเสนอประธานคณะอนุกรรมการฯพิจารณาขอความเห็นที่สองจากอนุกรรมการทบทวนฯคนอื่น

หากอนุกรรมการทบทวนฯคนที่สองยังคงยืนตาม ให้ประธานฯนำเข้าสู่การพิจารณาของกรรมการจริยธรรมเกี่ยวกับการทดลองต่อผู้เข้าร่วมการศึกษาตัดสิน โดยหัวหน้าโครงการวิจัยฯชี้แจง/ไม่ชี้แจงด้วยตนเอง

4 **ประเภทโครงการวิจัยฯ** (สามารถตรวจเลือกได้มากกว่าหนึ่งข้อ)

☒ 4.1. โครงการเดี่ยว (research project)

☐ 4.2. โครงการชุด (research program)

☐ 4.3. โครงการระหว่างภาควิชา/คณะภายใน (interdepartment, interfaculty)

☐ 4.4. โครงการศึกษาหลายศูนย์ (multicenter)

5 **ระดับการนำไปใช้ประโยชน์** (โปรดตรวจเลือกเพียงข้อเดียว)

☐ 5.1. ระดับทำด้วย (me too) เหมือนต่างประเทศ **และ** เคยศึกษาในประเทศไทย หมายถึง เหมือน/คล้ายคลึงกับการศึกษาในต่างประเทศ และมีการศึกษาในคำถาม/หัวข้อวิจัยนี้ในประเทศไทยมาแล้วจำนวนหนึ่ง

☒ 5.2. ระดับทำด้วย (me too) เหมือนต่างประเทศ **แต่** ไม่เคยศึกษาในประเทศไทย หมายถึง เหมือน/คล้ายคลึงกับการศึกษาในต่างประเทศ แต่ไม่มีการศึกษาในคำถาม/หัวข้อวิจัยนี้ในประเทศไทยมาก่อน

☐ 5.3. ระดับทำดีกว่า (me better) หมายถึง เหมือน/คล้ายคลึงกับการศึกษาในต่างประเทศ **แต่** มีการประยุกต์/ดัดแปลง/ปรับเปลี่ยนใน **เชิงใช้ประโยชน์** ให้ดียิ่งขึ้น

☐ 5.4. ระดับทำเป็นคนแรก (me first) หมายถึง เป็นการศึกษาคำถาม/หัวข้อวิจัยที่ **ไม่เคย** มีการศึกษามาก่อนในต่างประเทศ

6 **ชื่อหัวหน้าโครงการวิจัยฯ ชื่อผู้ร่วมวิจัย** **สัดส่วนการวิจัย** (ร้อยละ)

6.1. หัวหน้าโครงการ

นพ.อรรคพล ตั้งสัตยาธิษฐาน 50%

ตำแหน่ง แพทย์ประจำบ้าน

ภาควิชาจักษุวิทยา คณะแพทยศาสตร์ มหาวิทยาลัยสงขลานครินทร์

6.2. ผู้ร่วมวิจัย

รศ.พญ.สุภาภรณ์ เต็งไตรสรณ์ 50%

ตำแหน่ง อาจารย์

ภาควิชาจักษุวิทยา คณะแพทยศาสตร์ มหาวิทยาลัยสงขลานครินทร์

---

7 **แหล่งทุนภายนอกที่ได้รับ จำนวนเงินอุดหนุนที่ได้รับ (ถ้าเกี่ยวข้อง)**

-----

8 **บทนำ** เสมือนเป็นต้นฉบับพร้อมตีพิมพ์ ประกอบด้วย ปัญหา รายละเอียดของปัญหา และคำถามวิจัย (เป็นภาษาอังกฤษความยาว 300-500 คำ พร้อมระบุเอกสารอ้างอิง ใช้เลข (1) ตัวยก เมื่ออ้างเป็นครั้งแรก อ้างถัดไปใช้เลข (2), (3), (4) ตัวยก โดยลำดับ)

หมายเหตุ ในกรณีที่มีข้อจำกัด อนุโลมให้ทำเป็นภาษาไทย

ความสำคัญและที่มาของโครงการวิจัย (Background and Rationale)

Currently, diagnosis method and giving information of stage of **strabismus** had many ways such as alternate prism cover test, Hirschberg's test and Krimsky test seeing that the most standard

method was alternate prism cover test in which this diagnosis needed to be professional in ophthalmology (orthoptist), taking a long time to diagnosis, depending on the special device (prism) and patients were required to cooperate in the screening. The survey found that most hospitals of Thailand, locations especially were in rural area, were no professional in ophthalmology (orthoptist) including diagnostic equipments. Besides, heavy work of ophthalmologists therefore was not able to have an eye examination as alternate prism cover test by themselves.

Hirschberg's test was a simple screening that can be done quickly without the special equipment. Previous time, there was no more precision, and then the researcher therefore had invented digital camera that was an equipment with availability and affordable. This was improved by taking a photograph to measure reflection on corneal surface that caused Hirschberg's test more accuracy as well.

From the study showed that usage of **corneal light reflex** test by taking a photograph to calculate amount of changed millimeters were compared with the angle of deviation of strabismus and the standard method as alternate prism cover test that how many more relative values or less? The former study was researched within general public found that the angle of deviation of strabismus was from usage of **corneal light reflex** test by taking a photograph in strabismic patients compared with alternate prism cover test as 20.89 prism diopters/millimeter of corneal light reflex by a few of different values from alternate prism cover test (less than 5 prism diopters).

For such reason, this was source of the research to compare the angle of deviation of strabismus by **corneal light reflex** test and alternate prism cover test into real strabismic patients. The researcher hoped that the results can be applied to help diagnosis method and giving information of stage of **strabismus**. Particularly, most hospitals were without professional in ophthalmology. Otherwise, this can reduce ophthalmologist's heavy work because the method was very simple and the equipment was affordable. In addition to this can also be helped to reduce travel burden for patients in rural setting.

คำถามของการวิจัย (Research questions)

How about the comparison an angle of deviation from corneal light reflex photographs with Alternate prism cover test in strabismic patients?

วัตถุประสงค์ของโครงการวิจัย (Objectives)

1. (Main) To compare degree of an angle of deviation from the corneal light reflex photographs with alternated prism cover test in strabismic patients.

2. Expect data and methodology for ophthalmologist would be useful a conventional orthoptic test either at diagnosis, preoperative evaluation and following up the strabismic patients in rural setting.

9 **วัสดุและวิธีการ** เสมือนเป็นต้นฉบับพร้อมตีพิมพ์ ประกอบด้วย แหล่งที่มาของข้อมูล ประชากร กลุ่มตัวอย่าง วิธีการเลือกกลุ่มตัวอย่าง วิธีการรวบรวมข้อมูล การใช้เครื่องมือในการวิจัย และวิธีการวิเคราะห์ข้อมูล หรือใช้หลักสถิติมาประยุกต์

(เป็นภาษาอังกฤษความยาว 300-500 คำ พร้อมระบุเอกสารอ้างอิง ใช้เลข (1) ตัวยก เมื่ออ้างเป็นครั้งแรก อ้างถัดไปใช้เลข (2), (3), (4) ตัวยก โดยลำดับ)
หมายเหตุ ในกรณีที่มีข้อจำกัด อนุโลมให้ทำเป็นภาษาไทย

ลักษณะงานวิจัย (design)

- Analytic Prospective study.

แหล่งที่มาของข้อมูล

- Photographs from digital camera / orthoptic test

ประชากรกลุ่มตัวอย่าง (subjects)

Strabismic patients in Songklanakarin hospital. The total sample size required for this study is 53.

From Sample size calculation formula for correlation

$$N = \left[Z_{\alpha/2} + Z_{\beta} / C(r) \right]^2 + 3$$

When $Z_{\alpha/2}$ = **Proportion of type I error**

$C(r)$ = **Log_e { (1+r)/(1-r) }**

α **= 0.05** β **= 0.2** r **= 0.4**

เกณฑ์การคัดเลือก (Inclusion criteria)

1. Horizontal strabismic subjects.
2. Subject older than 5 years old.
3. Volunteer to participate in this study with informed consent.
4. Cooperate in an eye examination.

เกณฑ์คัดออก (Exclusion criteria)

1. Cyclovertical strabismus
2. Amblyopia
3. Accomodative esotropia
4. Paralytic strabismus
5. Strabismus has had previous strabismic surgery
6. Abnormal of ocular surface such as corneal diseases may be disturb corneal light reflex including pterygium

วิธีการรวบรวมข้อมูล

เมื่อโครงการผ่านความเห็นชอบจากคณะกรรมการจริยธรรม และได้รับการเซ็นต์ยินยอมเข้าร่วมโครงการวิจัยแล้วมีวิธีการดำเนินงานวิจัยดังนี้

Methods : The participants history and eye examination will be assessed and recorded including age, gender, presenting symptoms, duration of symptoms, underlying disease, history of eye surgery and etiology.

1. The study enrolled people who met the inclusion criteria.
2. Informed consent will be obtained from each subject prior to participation in the study.
3. Subject will sit at 1 m, 4 m from a screen and labeled with only one central target.
4. A total of 4 numbers are obtained in each Subject and each distance from screen, following to
   4.1 Subjects will be occluded one eye with a millimeter ruler and the other eye fix to central target on the screen.
   4.2 Subjects will be occluded anothers eye with a millimeter ruler and the other eye fix to central target on the screen.
   4.3 Subjects will be opened both eye with a millimeter ruler and the other eye fix to central target on the screen
   4.4 Subjects will be opened both eye with a millimeter ruler and the anothers eye fix to central target on the screen
5. A flash camera, aligned with the central target, will record the position of the corneal reflex.
6. From the photographs recorded, the distance from the corneal reflex to the center and nasal limbus will be measured with millimeter ruler scale in each photograph.
7. Data obtained from subjects in each direction of gaze will be analysed statistically.

ขั้นตอนดำเนินการวิจัย

ผู้วิจัยดำเนินการคัดเลือกผู้ป่วยตาเขตามเกณฑ์ที่กำหนดในวันที่ผู้ป่วยมาถึงโรงพยาบาล โดยผู้วิจัยเข้าพบผู้ป่วยทีละคน เพื่อแนะนำตนเองและบอกจุดประสงค์ในการวิจัย ให้ผู้ป่วยลงนามยินยอมในหนังสือแสดงความยินยอมพร้อมทั้งเก็บข้อมูลดังนี้

ข้อมูลพื้นฐาน ได้แก่ อายุ เพศ ประวัติการคลอด ประวัติการผ่าตัดตา

ข้อมูลประวัติโรคตาเข และโรคอื่นที่เกี่ยวข้อง

ข้อมูลตรวจร่างกายทางจักษุวิทยา และการวัดค่าสายตา

เครื่องมือที่ใช้ในการตรวจ ได้แก่ Slit lamp microscopy, prism และ camera (ดัง Flow Chart)

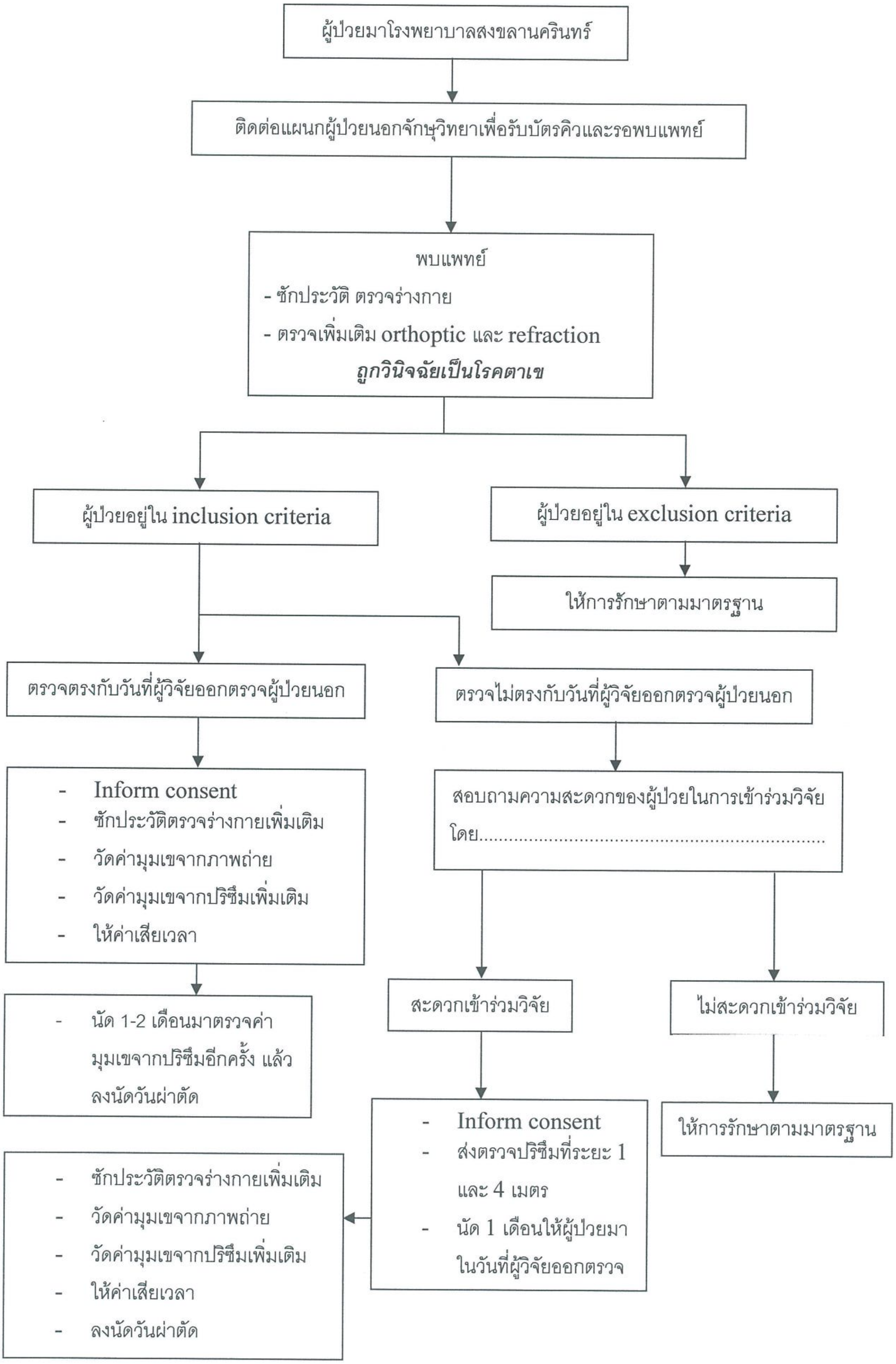
ผู้ป่วยมาโรงพยาบาลสงขลานครินทร์
ติดต่อแผนกผู้ป่วยนอกจักษุวิทยาเพื่อรับบัตรคิวและรอพบแพทย์
พบแพทย์
- ซักประวัติ ตรวจร่างกาย
- ตรวจเพิ่มเติม orthoptic และ refraction
ถูกวินิจฉัยเป็นโรคตาเข
ผู้ป่วยอยู่ใน inclusion criteria
ผู้ป่วยอยู่ใน exclusion criteria
ให้การรักษาตามมาตรฐาน
ตรวจตรงกับวันที่ผู้วิจัยออกตรวจผู้ป่วยนอก
ตรวจไม่ตรงกับวันที่ผู้วิจัยออกตรวจผู้ป่วยนอก
- Inform consent
- ซักประวัติตรวจร่างกายเพิ่มเติม
- วัดค่ามุมเขจากภาพถ่าย
- วัดค่ามุมเขจากปริซึมเพิ่มเติม
- ให้ค่าเสียเวลา
สอบถามความสะดวกของผู้ป่วยในการเข้าร่วมวิจัย
โดย..
- นัด 1-2 เดือนมาตรวจค่ามุมเขจากปริซึมอีกครั้ง แล้วลงนัดวันผ่าตัด
สะดวกเข้าร่วมวิจัย
ไม่สะดวกเข้าร่วมวิจัย
- Inform consent
- ส่งตรวจปริซึมที่ระยะ 1 และ 4 เมตร
- นัด 1 เดือนให้ผู้ป่วยมาในวันที่ผู้วิจัยออกตรวจ
ให้การรักษาตามมาตรฐาน
- ซักประวัติตรวจร่างกายเพิ่มเติม
- วัดค่ามุมเขจากภาพถ่าย
- วัดค่ามุมเขจากปริซึมเพิ่มเติม
- ให้ค่าเสียเวลา
- ลงนัดวันผ่าตัด

10 **เอกสารอ้างอิง** (ใช้ระบบ Vancouver) เรียงลำดับเอกสารอ้างอิงในบทนำ วัสดุและวิธีการ

1. Patrick A. Derespinis. Calibration of Hirschberg test photographs under clinical conditions. Ophthalmology 1989 January; 96:944-949.

2. Scott E. Brodie. Photographic Calibration of the Hirschberg test, New york hospital cornell medical center. Invest Ophthalmol Vis Sci 1985 November; 28:736-742.

3. Miller JM, Mellinger M, Greivenkemp J, Simons K. Videographic Hirschberg measurement of simulated strabismic deviations. Investigative Ophthalmology and Visual Science. 1993 October; 34 (11):3220-3229.

4. Barry JC, Backes A. Limbal versus pupil center for ocular alignment measurement with corneal reflexes, RWTH Aachen university, Germany. Ivest Ophthalmol Vis Sci. 1997 November; 38(12): 2597-607.

5. Brodie SE. Photographic calibration of the hirschberg test. Ophthalmol Vis Sci 1987 April; 28(4):736-42

6. Barry JC. Hirschberg erred here: the correct angle factor is 12 degrees pro mm corneal reflex decentration. Geometric optical analysis of various methods in strabismometry. Klin Monbl Augenheilkd. 1999 August; 215 (2): 104-13.

7. Romano PE. Individual case photogrammetric calibration of the Hirschberg Ratio (HR) for corneal light reflection test strabometry. Binocular Vision Strabismus Quarterly. 2006; 21(1):45-6.

11 **ระยะเวลาที่ทำการวิจัย**

วันที่ 1 เดือน เมษายน พ.ศ. 2556 ถึง วันที่ 31 เดือน ธันวาคม พ.ศ. 2557

- เขียน proposal และส่งคณะกรรมการพิจารณาจริยธรรมด้านวิจัย ธ.ค.2555 – มี.ค.2556
- เก็บรวบรวมข้อมูล เม.ย.2556 – ธ.ค.2556
- วิเคราะห์ข้อมูล ม.ค.2557 – มี.ค.2557
- เขียนรายงานและนำเสนองานวิจัย เม.ย.2557 – มิ.ย.2557

12 **ประสงค์ขอรับเงินอุดหนุนการวิจัยจากกองทุนวิจัย คณะแพทยศาสตร์**

รายละเอียดงบประมาณ (ระบุ)

- ค่าตอบแทนเจ้าหน้าที่วัดมุมเขและช่างถ่ายภาพ (ผู้ป่วย 53 คน x 100 บาท x 2)	10,600.- บาท
- ค่าบันทึกข้อมูลวิจัยของเจ้าหน้าที่ฝ่ายคอมพิวเตอร์ (ผู้ป่วย 53 คน x 100 บาท)	5,300.- บาท
- ค่าเสียเวลาผู้ป่วย (ผู้ป่วย 53 คน x 500 บาท)	26,500.- บาท
- ค่าถ่ายสำเนาเอกสารและอุปกรณ์เก็บข้อมูล	1,000.- บาท
- ค่าวิเคราะห์ข้อมูล	2,000.- บาท
- ค่าจัดทำรายงานวิจัย	2,000.- บาท
รวมจำนวนเงิน	47,400.- บาท

(สี่หมื่นเจ็ดพันสี่ร้อยบาท)

(เสนอขอรับทุนอุดหนุนโครงการวิจัยจากกองทุนวิจัย ทุนอุดหนุนวิจัยสุขภาพ)

**หมายเหตุ orthoptic, refraction และ eye exam ให้คิดตามสิทธิการรักษาของผู้ป่วย

13 **คำรับรองจากหัวหน้าโครงการวิจัยฯ**

ข้าพเจ้าส่งข้อเสนอแบบฟอร์ม จธ 02 (2554) บริบาลผู้ป่วย และสิ่งที่ต้องเสนอพร้อมข้อเสนอโครงการวิจัยฯ จำนวน 3 ชุด

พร้อมแผ่นบันทึกจำนวน 1 แผ่น ประกอบด้วยไฟล์ของ

1. แบบฟอร์ม จธ 02 (2554) บริบาลผู้ป่วย
2. **แบบเอกสารแนะนำผู้ป่วย/ลงนามยินยอม (3.1.1. และ 3.1.2.)** (ถ้าเกี่ยวข้อง)
3. **แบบฟอร์ม จธ 06 (2254) เก็บตัวอย่างชีวภาพตรวจ ณ ต่างประเทศ** (ถ้าเกี่ยวข้อง)
4. **แบบฟอร์ม จธ 07 (2554) เก็บตัวอย่างชีวภาพเพื่อศึกษาวิจัยในอนาคต** (ถ้าเกี่ยวข้อง)
5. แบบลงข้อมูล
6. อัตตประวัติหัวหน้าโครงการวิจัยฯ

ข้าพเจ้าขอรับรองว่า ข้อความในข้อเสนอโครงการวิจัยฯและสิ่งที่ต้องเสนอพร้อมข้อเสนอโครงการวิจัยฯมีความถูกต้องเป็นจริง หากมีการปรับปรุงแก้ไขเอกสารเกี่ยวกับการวิจัย (revised research document) เป็นต้นว่า การแก้ไขแบบแผนการดำเนินการวิจัย (protocol amendment) การทำให้รายละเอียดเกี่ยวกับผลิตภัณฑ์มีข้อมูลทันสมัย (update investigator brochure) และการแก้ไขหนังสือยินยอมและเอกสารแนะนำผู้ป่วย (revised consent form/information sheet) ข้าพเจ้าจะแจ้งให้คณะอนุกรรมการพิจารณาจริยธรรมด้านวิจัยฯ ทราบ

ข้าพเจ้ามี/จัดให้มีกระบวนการลงนามยินยอมอย่างเต็มที่ และ ลงนามใบยินยอมของโครงการวิจัยฯ ตามประเภทโครงการวิจัยฯ หรือ

ข้าพเจ้ามี/จัดให้มีกระบวนการยินยอมด้วยวาจาอย่างเต็มที่ และ ลงนามใบยินยอมของโรงพยาบาล (อยู่โรงพยาบาล หรือ การผ่าตัดที่รุกล้ำ) ตามประเภทโครงการวิจัยฯ

ข้าพเจ้ามี/จัดให้มีกระบวนการ การกำกับ และตรวจสอบตามหลักเกณฑ์การบริบาลผู้ป่วยรวมถึงติดตาม/ดูแลด้านความปลอดภัยของผู้ป่วย

ข้าพเจ้าเป็นผู้รายงานความคืบหน้าโครงการวิจัยฯทุก 6 เดือน นับตั้งแต่วันที่ได้รับการรับรองด้านจริยธรรม หรือ ทุกครั้งที่เบิกจ่ายเงินอุดหนุนงวดต่อไป

ข้าพเจ้าเป็นผู้รายงานเหตุการณ์ไม่พึงประสงค์ร้ายแรงของผู้ป่วย ผู้เข้าร่วมโครงการวิจัยฯนี้ ให้หัวหน้าภาควิชา/หน่วยงาน และ ผู้อำนวยการโรงพยาบาลสงขลานครินทร์ทราบ ด้วยแบบรายงานเหตุการณ์ (incidence report) ของโรงพยาบาลฯ พร้อมสำเนาให้ประธานอนุกรรมการพิจารณาจริยธรรมด้านวิจัยฯ ทราบ

ข้าพเจ้ามี/จัดให้มีกระบวนการติดตาม/ชำระความถูกต้องของข้อมูล

ในกรณีเคลื่อนย้ายสิ่งส่งตรวจที่ได้มาจากผู้ป่วยในโครงการวิจัยฯ ทั้งเป็น/ไม่เป็นการบริบาลผู้ป่วย เช่น เลือด สารคัดหลั่ง เนื้อเยื่อและอวัยวะ เพื่อรอตรวจ/ตรวจทางห้องปฏิบัติพิเศษ ณ ต่างประเทศ ข้าพเจ้าต้องให้เสนอขอความเห็นและอนุมัติจากคณะกรรมการฯก่อน ในกรณีมีความคืบหน้าเกี่ยวกับผลการตรวจพิเศษทางห้องปฏิบัติการ ข้าพเจ้าเป็นผู้รายงานให้คณะกรรมการฯทราบ

ข้าพเจ้าขอให้คำมั่นและปฏิบัติตามจรรยาบรรณนักวิจัยฯ ซึ่งประกาศโดยสภาวิจัยแห่งชาติ

เมื่อเสร็จสิ้นโครงการวิจัยฯ ในกรณีรับทุนอุดหนุนจากแหล่งทุนภายนอก ข้าพเจ้าเป็นผู้ทำบทคัดย่อ (abstract) เสนอฝ่ายวิจัย ส่วนกรณีรับทุนอุดหนุนจากกองทุนวิจัย คณะแพทยศาสตร์ ข้าพเจ้าเป็นผู้ทำต้นฉบับพร้อมตีพิมพ์ (manuscript) แบบบทความดั้งเดิม (original article) **หรือ** บทความสั้น (short article) เสนอฝ่ายวิจัย

ลงชื่อ [signature]

(นายแพทย์อรรคพล ตั้งสัตยาธิษฐาน)

ตำแหน่ง หัวหน้าโครงการวิจัย

วันที่ 22 เดือน ก.พ. พ.ศ. 2556

หมายเหตุ ฝ่ายวิจัยยกเลิกการส่งรายงานวิจัยฉบับสมบูรณ์ ตามบันทึกที่ ม.อ. 351.7.2/2751 ลงวันที่ 18 สิงหาคม 2553 ให้หัวหน้าโครงการวิจัยฯพิจารณาเลือกส่งเฉพาะต้นฉบับพร้อมตีพิมพ์ ***ภาษาอังกฤษ*** เป็นลำดับความสำคัญแรก ส่วน ต้นฉบับพร้อมตีพิมพ์ ***ภาษาไทย*** เป็นลำดับความสำคัญที่สอง ประกอบด้วยชื่อเรื่องและเนื้อหา 5 ส่วน คือ บทนำ วัสดุและวิธีการ ผลลัพธ์ อภิปราย และเอกสารอ้างอิง

ต้นฉบับภาษาอังกฤษให้สะกดชื่อ คณะแพทยศาสตร์ มหาวิทยาลัยสงขลานครินทร์ให้ถูกต้อง คือ Faculty of Medicine, Prince of Songkla University เพื่อสิทธิในการขอรับรางวัลผลงานวิจัย ทั้งนี้ มหาวิทยาลัยไม่อนุมัติเงินอุดหนุนค่าตีพิมพ์/รางวัลผลวิจัยตีพิมพ์ หากสะกดชื่อคณะแพทยศาสตร์และมหาวิทยาลัยเป็นอื่น

ในกรณีที่มีข้อจำกัด ไม่สามารถเลือกส่งเป็นต้นฉบับพร้อมตีพิมพ์ ให้หัวหน้าโครงการวิจัยพิจารณาเลือกส่งเป็น บทคัดย่อ เป็น ***ภาษาอังกฤษ*** **หรือ** ***ภาษาไทย*** ประกอบเนื้อหา 5 ส่วนเช่นเดียวกับต้นฉบับพร้อมตีพิมพ์

ทั้งต้นฉบับพร้อมตีพิมพ์ **หรือ** บทคัดย่อ มีประโยชน์ต่อ **ผลงานวิจัยตีพิมพ์ หรือ ผลงานวิจัยที่นำไปใช้ประโยชน์** ตามตัวบ่งชี้ที่ 5 และ 6 (สมศ)

14 **คำอนุมัติจากหัวหน้าภาควิชา/หน่วยงานหรือเทียบเท่า**

[signature]

ลงชื่อ

(ผศ.นพ.ธวัช ต้นติสารศาสน์)

ตำแหน่ง หัวหน้าภาควิชาจักษุวิทยา

วันที่ 22 เดือน กพ พ.ศ. 2556
