# Supplementary material for: The reliability of the angle of deviation measurement from the Photo-Hirschberg tests and Krimsky tests
Source: PLoS One. 2021 Dec 1;16(12):e0258744. doi: 10.1371/journal.pone.0258744 (PMC8635364; doi:10.1371/journal.pone.0258744)
Supplement: S3 File — (PDF) [file pone.0258744.s003.pdf]

Form of a proposal for a research project

Subcommittee on Ethical Research on Patient Care, Specimens and Medical Social Sciences

Committee for Screening and Considering Research Grants - Considering Subsidized Research Fund

(If relevant)

1. Title:

The comparison an angle of deviation from photographs with alternate prism cover test in strabismic patients

2. Keywords

Hirschberg test, alternate prism cover test, horizontal strabismus, angle of deviation, photographs

3. Design study

3.1: low risk project and no standard management in Thailand

3.1.2 A study of new surgical techniques / methods / devices with results of previous studies in other countries.

What must be submitted together with the research proposal

- Relevant / similar in-country study results (1 to 2 abstracts)
- Have / provide a full consent signing process (3.1.1. and 3.1.2)
- Detailed consent form according to the patient introduction / consent form (3.1.1. and 3.1.2.), avoiding the use of doctor / English terminology as much as possible
- Data record
- Be aware of section 13 about reporting serious adverse events.

4. Design project:

4.1 Research project

5. Level of utilization:

5.2 The degree to do with (me too) is like a foreign country but has never studied in Thailand. But there has not been any previous study of this research question / topic in Thailand.

6. **List and duties of the research team** **proportion**

6.1. Project leader

Instructor

Assoc.Prof.Supaporn Tengtrisorn 50%

Department of Ophthalmology, Faculty of Medicine,

Prince of Songkla University

6.2. **Co-researcher**

Resident

Akkapol Tungsattayathitthan 50%

Department of Ophthalmology, Faculty of Medicine,

Prince of Songkla University

7. **External funds**: no

8. **Introduction**

Background and Rationale:

Currently, diagnosis method and giving information of stage of **strabismus** had many ways such as alternate prism cover test, Hirschberg's test and Krimsky test seeing that the most standard method was alternate prism cover test in which this diagnosis needed to be professional in ophthalmology (orthoptist), taking a long time to diagnosis, depending on the special device (prism) and patients were required to cooperate in the screening. The survey found that most hospitals of Thailand, locations especially were in rural area, were no professional in ophthalmology (orthoptist) including diagnostic equipments. Besides, heavy work of ophthalmologists therefore was not able to have an eye examination as alternate prism cover test by themselves.

Hirschberg's test was a simple screening that can be done quickly without the special equipment. Previous time, there was no more precision, and then the researcher therefore had invented digital camera that was an equipment with availability and affordable. This was improved by taking a photograph to measure reflection on corneal surface that caused Hirschberg's test more accuracy as well.

From the study showed that usage of **corneal light reflex** test by taking a photograph to calculate amount of changed millimeters were compared with the angle of deviation of strabismus and the standard method as alternate prism cover test that how many more relative values or less? The former study was researched within general public found that the angle of deviation of strabismus was from usage of **corneal light reflex** test by taking a photograph in strabismic patients compared with alternate prism cover test as 20.89 prism

diopters/millimeter of corneal light reflex by a few of different values from alternate prism cover test (less than 5 prism diopters).

For such reason, this was source of the research to compare the angle of deviation of strabismus by **corneal light reflex** test and alternate prism cover test into real strabismic patients. The researcher hoped that the results can be applied to help diagnosis method and giving information of stage of **strabismus**. Particularly, most hospitals were without professional in ophthalmology. Otherwise, this can reduce ophthalmologist's heavy work because the method was very simple and the equipment was affordable. In addition to this can also be helped to reduce travel burden for patients in rural setting.

Research questions:

How about the comparison an angle of deviation from corneal light reflex photographs with Alternate prism cover test in strabismic patients?

Objectives:

1. (Main) To compare degree of an angle of deviation from the corneal light reflex photographs with alternated prism cover test in strabismic patients.
2. Expect data and methodology for ophthalmologist would be useful a conventional orthoptic test either at diagnosis, preoperative evaluation and following up the strabismic patients in rural setting.

9. **Material and method:**

Design: Analytic Prospective study.

Source of data: Photographs from digital camera / orthoptic test

Subjects:

Strabismic patients in Songklanakarin hospital. The total sample size required for this study is 53. From Sample size calculation formula for correlation

$$N = \left[Z_{\alpha/2} + Z_{\beta} / C(r) \right]^2 + 3$$

When $Z_{\alpha/2}$ = **Proportion of type I error**

$C(r)$ = **$\mathbf{Log_e}$ { (1+r)/(1-r) }**

α = **0.05** β = **0.2** r = **0.4**

Inclusion criteria

1. Horizontal strabismic subjects.
2. Subject older than 5 years old.
3. Volunteer to participate in this study with informed consent.
4. Cooperate in an eye examination.

Exclusion criteria

1. Cyclovertical strabismus
2. Amblyopia
3. Accomodative esotropia
4. Paralytic strabismus
5. Strabismus has had previous strabismic surgery
6. Abnormal of ocular surface such as corneal diseases may be disturb corneal light reflex including pterygium

Methods : The participants history and eye examination will be assessed and recorded including age, gender, presenting symptoms, duration of symptoms, underlying disease, history of eye surgery and etiology.

1. The study enrolled people who met the inclusion criteria.
2. Informed consent will be obtained from each subject prior to participation in the study.
3. Subject will sit at 1 m, 4 m from a screen and labeled with only one central target.
4. A total of 4 numbers are obtained in each Subject and each distance from screen, following to

   4.1 Subjects will be occluded one eye with a millimeter ruler and the other eye fix to central target on the screen.

   4.2 Subjects will be occluded anothers eye with a millimeter ruler and the other eye fix to central target on the screen.

   4.3 Subjects will be opened both eye with a millimeter ruler and the other eye fix to central target on the screen

   4.4 Subjects will be opened both eye with a millimeter ruler and the anothers eye fix to central target on the screen

      1.A flash camera, aligned with the central target, will record the position of the corneal reflex.

      2.From the photographs recorded, the distance from the corneal reflex to the center and nasal limbus will be measured with millimeter ruler scale in each photograph.

      3.Data obtained from subjects in each direction of gaze will be analysed statistically.

10. Reference:

1. Patrick A. Derespinis. Calibration of Hirschberg test photographs under clinical conditions. Ophthalmology 1989 January; 96:944-949.
2. Scott E. Brodie. Photographic Calibration of the Hirschberg test, New york hospital cornell medical center. Invest Ophthalmol Vis Sci 1985 November; 28:736-742.
3. Miller JM, Mellinger M, Greivenkemp J, Simons K. Videographic Hirschberg measurement of simulated strabismic deviations. Investigative Ophthalmology and Visual Science. 1993 October; 34 (11):3220-3229.
4. Barry JC, Backes A. Limbal versus pupil center for ocular alignment measurement with corneal reflexes, RWTH Aachen university, Germany. Ivest Ophthalmol Vis Sci. 1997 November; 38(12): 2597-607.
5. Brodie SE. Photographic calibration of the hirschberg test. Ophthalmol Vis Sci 1987 April; 28(4):736-42.
6. Barry JC. Hirschberg erred here: the correct angle factor is 12 degrees pro mm corneal reflex decentration. Geometric optical analysis of various methods in strabismometry. Klin Monbl Augenheilkd. 1999 August; 215 (2): 104-13.
7. Romano PE. Individual case photogrammetric calibration of the Hirschberg Ratio (HR) for corneal light reflection test strabometry. Binocular Vision Strabismus Quarterly. 2006; 21(1):45-6.

11. Research period: 1 April 2013 to 31 October 2019

- proposal design and EC process 10/2012 - 03/2013
- data collection 04/2013 - 12/2015
- data analysis 01/2016 - 12/2016
- Report and presentation 01/2017 - 10/2019

12. Faculty of Medicine fund: 47,400 baths

13. Certification from the research project:

I submit a proposal form JT 02 (2011) nourishment of the patient and things that need to be submitted together with the research proposal in the amount of 3 copies.

A memory card contains the files of

1. A proposal form JT 02 (2011) nourishment of the patient
2. Patient introduction / consent form (3.1.1. and 3.1.2.)
3. JT 06 (2254) biological sample collection for inspection abroad (if relevant)

4. JT 07 (2011) collection of biological samples for future research studies. (If relevant)
5. Record form
6. Profile of project Leader

I certify that The statements in the research proposal and the proposals that must be submitted together with the research proposal are accurate and truthful.

If there is an amendment to the research document such as revised research document, protocol amendment, update investigator brochure and revised consent form/information sheet, I will notify Research Ethics Subcommittee.

I set up a fully informed consent signing process and sign the consent form of the research project according to the type of research project.

I have / set up a supervisory and review process in accordance with the patient care criteria, including Follow / take care of patient safety

I am going to progress report of the research program every 6 months starting from the date of ethical certification or each time the next subsidy disbursement is disbursed.

I am going to report a serious adverse event, participants in this research project to the head of department / department and the director of Songklanagarin hospital with incidence report form together with a copy to the chairman of the ethics review subcommittee on research.

I have established a procedure for tracking / payment of information accuracy in the case of transferring specimens obtained from patients in the trial such as blood, secretion, tissue and organs for examination at a special laboratory abroad. I must seek an opinion and approval from the Board. In the event that there is progress regarding the special laboratory examination results I will report to the Board of Directors for acknowledgment

I hereby make a commitment and abide by the Researcher's Code of Conduct which was announced by the National Research Council. Upon completion of the research project In the case of receiving funding from external sources I am the author of the abstract (abstract) proposing to research department. In the case of receiving funding from the research fund Faculty of Medicine I will write a manuscript, original article or short article, proposed to research department.

sign --

(**Akkapol Tungsattayathitthan**)

----

14. Approval from the head of the department

sign --

(Assist. Prof.Tawat Tantisarasart)
