# Supplementary material for: The reliability of the angle of deviation measurement from the Photo-Hirschberg tests and Krimsky tests
Source: PLoS One. 2021 Dec 1;16(12):e0258744. doi: 10.1371/journal.pone.0258744 (PMC8635364; doi:10.1371/journal.pone.0258744)
Supplement: S4 File — (PDF) [file pone.0258744.s004.pdf]

Faculty of Medicine, Prince of Songkla University

This document is to certify that

REC Number:	57-0117-02-7
Project entitled:	The accuracy of strabismic measurement with Kimsky test
Principle Investigator:	Assoc.Prof.Supaporn Tengtrisorn, M.D.
Affiliation:	Department of Ophthalmology, Faculty of Medicine, Prince of Songkla University
Sub-Investigator:	1. Mrs.Somporn Bhurachokwiwat
Affiliation:	Department of Ophthalmology, Faculty of Medicine, Prince of Songkla University
Sub-Investigator:	2. Miss.Srirabay Chouyjan
Affiliation:	Department of Ophthalmology, Faculty of Medicine, Prince of Songkla University
Sub-Investigator:	3. Akkapol Tungsatthayathithan, M.D.
Affiliation:	Department of Ophthalmology, Faculty of Medicine, Prince of Songkla University
Sub-Investigator:	4. Asst.Prof.Pennee Singha, M.D.
Affiliation:	Department of Ophthalmology, Faculty of Medicine, Prince of Songkla University

Document acceptance:

1. Submission form version 1.0 date 26 March 2014
2. Clinical record form version 1.0 date 26 March 2014
3. Curriculum Vitae

have been reviewed by the Research Ethics Committee is in full compliance with the Declaration of Helsinki and the International Conference on Harmonization in Good Clinical Practice (ICH-GCP) Guidelines. Please submit the progress report every 12 months.

....

(Assoc. Prof.Boonsin Tangtrakulwanich)

Chairman of Research Ethics Committee,

Date of approval: July 8, 2014

Date of expiration: July 7, 2015

Office of Human Research Ethics Committee
Faculty of Medicine Prince of Songkla University
15 Karnjanavanit Rd. Hat YaiSongkhla 90110
Tel. +66 7445-1149, +667445-1157
Fax 66 7421-2900
