# Supplementary material for: The reliability of the angle of deviation measurement from the Photo-Hirschberg tests and Krimsky tests
Source: PLoS One. 2021 Dec 1;16(12):e0258744. doi: 10.1371/journal.pone.0258744 (PMC8635364; doi:10.1371/journal.pone.0258744)
Supplement: S5 File — (PDF) [file pone.0258744.s005.pdf]

แบบเสนอโครงการวิจัยทางด้านคลินิก (Clinical Trial)

**ข้อมูลที่ให้ระบุในแบบเสนอโครงการวิจัย มีจุดประสงค์เพื่อให้ reviewer และคณะกรรมการจริยธรรม สามารถ ประเมินคุณภาพของโครงการวิจัย กรุณาเขียนให้ตรงตามหัวข้ออย่างชัดเจนและครบถ้วน เนื่องจากเนื้อหาที่ระบุมีความสำคัญต่อความรวดเร็วในการประเมินคุณภาพของโครงการวิจัย

1.**ชื่อโครงการ** (ชัดเจนเหมาะสม)

- ชื่อภาษาไทย "ความแม่นยำในการวัดมุมเขด้วยวิธี kimsky""

  คำสำคัญ (ภาษาไทย) แสงสะท้อนผิวกระจกตา, ปริซึม, ตาเข, มุมเข, ภาพถ่าย

- ชื่อภาษาอังกฤษ "The accuracy of strabismic measurement with Kimsky test."

  Keywords (ภาษาอังกฤษ) Kimsky test, alternate prism cover test, horizontal strabismus, angle of deviation

2.**รายชื่อและหน้าที่ของคณะผู้วิจัย**

หัวหน้าโครงการ

รศ.พญ.สุภาภรณ์ เต็งไตรสรณ์ สัดส่วนการวิจัย 50%

SUPAPORN TENGTRISORN, MD

ตำแหน่ง อาจารย์แพทย์ ภาควิชาจักษุวิทยา

คณะแพทยศาสตร์ มหาวิทยาลัยสงขลานครินทร์

หน้าที่ Proposal development

Data collection

Data analysis

Manuscript writing

ผู้ร่วมวิจัย

สมพร บูรโชควิวัฒน์ สัดส่วนการวิจัย 15%

SOMPORN BHURACHOKWIWAT

ตำแหน่ง orthoptist ภาควิชาจักษุวิทยา

คณะแพทยศาสตร์ มหาวิทยาลัยสงขลานครินทร์

หน้าที่ Collecting data

ผู้ร่วมวิจัย

ศรีระบาย ช่วยจันทร์ สัดส่วนการวิจัย 15%

SRIRABAY CHOUYJAN

ตำแหน่ง orthoptist ภาควิชาจักษุวิทยา

คณะแพทยศาสตร์ มหาวิทยาลัยสงขลานครินทร์

หน้าที่ Collecting data

ผู้ร่วมวิจัย

นพ.อรรคพล ตั้งสัตยาธิษฐาน สัดส่วนการวิจัย 10%

AKKAPOL TUNGSATTHAYATHITHAN, MD

ตำแหน่ง แพทย์ประจำบ้าน ภาควิชาจักษุวิทยา

คณะแพทยศาสตร์ มหาวิทยาลัยสงขลานครินทร์

หน้าที่ Collecting data

ผู้ร่วมวิจัย

ผศ.พญ.เพ็นนี สิงหะ สัดส่วนการวิจัย 10%

PENNEE SINGHA, MD

ตำแหน่ง อาจารย์แพทย์ ภาควิชาจักษุวิทยา

คณะแพทยศาสตร์ มหาวิทยาลัยสงขลานครินทร์

หน้าที่ Manuscript writing

3. หลักการและเหตุผล

ตาเขเป็นความผิดปกติที่พบได้บ่อยและมีความสัมพันธ์กับพัฒนาการการมองเห็นเป็นอย่างมาก โดยเฉพาะอย่างยิ่งในเด็กซึ่งพัฒนาการเกิดขึ้นอย่างต่อเนื่องและรวดเร็ว หากสภาพตาเขเกิดในเด็กได้รับการตรวจพบรวดเร็วและให้การรักษาได้ทันท่วงที

ย่อมจะเป็นผลดีต่อการพัฒนาเป็นอย่างยิ่ง การตรวจหามุมเขที่ใช้ได้เหมาะสมที่สุดจะเป็นการตรวจด้วย kimsky test เนื่องจากไม่ต้องอาศัยความร่วมมือจากผู้ป่วยเด็กมากนัก แต่ขณะเดียวกันความแม่นยำก็อาจจะไม่ค่อยแน่นอนขึ้นกับความสามารถของผู้วัดซึ่งมีความแตกต่างกันได้มาก ดังนั้นหากจะดูความแม่นยำก็คงต้องเปรียบเทียบกับการตรวจด้วยวิธี alternate prism cover test ซึ่งจะตรวจได้ในผู้ป่วยที่ให้ความร่วมมือเท่านั้น ที่ผ่านมาคณะผู้วิจัยได้รับการอนุมัติให้ทำงานวิจัยเรื่อง “The comparison an angle of deviation from photographs with alternate prism cover test in strabismic patients.” ซึ่งจะเริ่มเก็บข้อมูลในวันที่ 1 ตุลาคมนี้ จึงขอเพิ่มเติมการตรวจมุมเขวิธี kimsky test ในกลุ่มผู้ป่วยเดียวกัน ปัญหานี้พบบ่อยในโรงพยาบาลสงขลานครินทร์และเรามีความพร้อมทั้งในด้านบุคลากรและทรัพยากรในการวิจัยครั้งนี้

4. วัตถุประสงค์และสมมติฐาน

เพื่อหาความสัมพันธ์ของการวัดมุมเขด้วยวิธี kimsky test และ alternate prism cover

5. การทบทวนวรรณกรรม

Joo KS และคณะได้ศึกษาหาความสัมพันธ์ของการวัดมุมเขด้วยวิธี Kimsky test ระยะไกล และ alternate prism cover ในผู้ป่วยตาเขเข้า 20 คน ตาเขออก 20 คน พบว่าได้ผลค่อนข้างดีเมื่อเทียบกับการวัดด้วยวิธี kimsky test ตามปกติ และมีประโยชน์ในกรณีที่ต้องการวัดมุมเขในผู้ป่วยที่ไม่ร่วมมือ

Choi RY และคณะได้ศึกษาความแม่นยำในการวัดมุมเขด้วยวิธี Hirschberg และ Kimsky ในจักษุแพทย์ด้านโรคตาเขจำนวน 16 คน โดยให้ประเมินมุมเขโดยวิธี Hirschberg จากภาพผู้ป่วยตาเข 4 คน พบว่า การประเมินด้วยวิธี Hirschberg พบว่าน้อยกว่าความเป็นจริง อย่างน้อย 10 prism diopter(PD) และมีแนวโน้มจะประเมินได้น้อยกว่าความเป็นจริงทั้งกลุ่มที่มีมุมเขมากและมุมเขน้อย ส่วนการวัดด้วยวิธี Kimsky โดยส่วนใหญ่ประเมินได้มากกว่าความเป็นจริง 10 PD ในผู้ป่วยอย่างน้อย 1 ราย และแสดงให้เห็นว่าไม่สามารถแยกความแตกต่างที่ต่างกัน 5 PD สรุปว่าทั้ง 2 วิธีขาดความแม่นยำในการวัดมุมเข เมื่อเทียบกับวิธี alternate prism covor

6. การออกแบบงานวิจัย

ตามโครงการวิจัย “The comparison an angle of deviation from photographs with alternate prism cover test in strabismic patients.” ซึ่งได้รับการอนุมัติการวิจัยแล้ว โดยเพิ่มเติมการวัดด้วย Kimsky เข้าไปก่อนการวัดด้วยวิธี alternate prism cover

7.การคัดเลือกกลุ่มตัวอย่าง

มีเกณฑ์การคัดเข้าและคัดออกจากโครงการอย่างเหมาะสม ตามโครงการวิจัย “The comparison an angle of deviation from photographs with alternate prism cover test in strabismic patients.” ซึ่งได้รับการอนุมัติการวิจัยแล้ว

8.การรักษา/วิจัย

การดำเนินงานวิจัยจะเริ่มด้วยการวัดมุมเขด้วยวิธี Kimsky test โดยผู้วัดมุมเขจะไม่ทราบมาก่อนว่าผู้ป่วยเคยได้รับการวัดมุมเขเท่าไหร่ และตามด้วยการถ่ายภาพและวัดมุมเขด้วยวิธี alternate prism cover test ตามโครงการวิจัย “The comparison an angle of deviation from photographs with alternate prism cover test in strabismic patients.” ซึ่งได้รับการอนุมัติการวิจัยแล้ว

9.การติดตามดูแลผู้ป่วย

ตามโครงการวิจัย “The comparison an angle of deviation from photographs with alternate prism cover test in strabismic patients.” ซึ่งได้รับการอนุมัติการวิจัยแล้ว

10.การประเมินผลตัวแปรที่ศึกษา

ตัวแปรในการศึกษาเป็น

มุมเขที่วัดด้วยวิธี kimsky test และมุมเขจากการวัดด้วยวิธี alternate prism cover test

11.กลุ่มตัวอย่าง

ตามโครงการวิจัย “The comparison an angle of deviation from photographs with alternate prism cover test in strabismic patients.” ซึ่งได้รับการอนุมัติการวิจัยแล้ว

12.การจัดการข้อมูล

- นำข้อมูลการตรวจ APCT และข้อมูลทั่วไปจากการศึกษา “The comparison an angle of deviation from photographs with alternate prism cover test in strabismic patients.”
- Orthoptis 1 คน ตรวจตาเขด้วยวิธี Kimsky ที่ห้องตรวจตาผู้ป่วยนอก โรงพยาบาลสงขลานครินทร์
- เปรียบเทียบความแตกต่างของการวัดมุมเข 2 วิธีว่ามีความสัมพันธ์เป็นอย่างไร

13.การวิเคราะห์ทางสถิติ

ใช้การวิเคราะห์หาความสัมพันธ์ระหว่างการตรวจตาเข วิธี Kimsky และ alternate prism cover test โดยใช้ Pearson’s Correlation

14.เกณฑ์ทางจริยธรรม

ตามโครงการวิจัย “The comparison an angle of deviation from photographs with alternate prism cover test in strabismic patients.” ซึ่งได้รับการอนุมัติการวิจัยแล้ว

15.ประโยชน์ที่จะได้รับจากการวิจัยนี้ในภาพรวมของระบบสาธารณสุขประเทศไทย

นำเสนอการวัดมุมเขที่แม่นยำในโรงพยาบาลที่ขาดแคลนบุคลากรทางจักษุวิทยา

16.งบประมาณ (อิงตามเกณฑ์ประกาศ คณะแพทย์จาก website หน่วยส่งเสริมฯ)

- ค่าบันทึกข้อมูลวิจัยของเจ้าหน้าที่ฝ่ายคอมพิวเตอร์ (ผู้ป่วย 53 คน x 100 บาท) 5,300 บาท
- ค่าถ่ายสำเนาเอกสารและอุปกรณ์เก็บข้อมูล 1,000 บาท
- ค่าวิเคราะห์ข้อมูล 3,000 บาท
- ค่าจัดทำรายงานวิจัย 2,000 บาท

รวมจำนวนเงิน 11,300.- บาท

(หนึ่งหมื่นหนึ่งพันสามร้อยบาท)

(เสนอขอรับทุนอุดหนุนโครงการวิจัยจากกองทุนวิจัย ทุนอุดหนุนวิจัยสุขภาพ)

**หมายเหตุ

- งบในส่วนการให้บริการเนื่องจากเก็บข้อมูลผู้ป่วยโครงการ "การเปรียบเทียบค่ามุมเขจากการใช้ภาพนิ่งวัดแสงสะท้อนผิวกระจกตา กับค่ามุมเขจากการวัดปริซึม ในผู้ป่วยตาเข"
- ขอเกลี่ยงบประมาณในทุกรายการ

17.เอกสารอ้างอิง

1. Joo KS,Koo H, Moon NJ. Measurement of strbismic angle using the distance krimsky test. Korean J Ophthalmol 2013 Aug;27(4):276-81.

2. Choi RY, Kushner BJ. The accracy of experienced strabismologists using the Hirschberg and Krimsky test. Ophthalmology 1998 Jul; 105(7):1301-6.

3. Thompson JT, Guyton DL. Ophthalmic prism. Measurement error and how to minimize them. Ophthalmology 1983;90(3):204-10.

18.ภาคผนวก

- มีแบบฟอร์มการบันทึกข้อมูล (case record form)
- มีวิธีการรายงาน adverse events และ adverse reactions ในแบบฟอร์ม (ตัวอย่างการเขียนดูจาก website หน่วยส่งเสริมฯ)
- มีแบบฟอร์มสรุป outcome ในตอนสุดท้าย เมื่อสิ้นสุดการติดตาม GCP3 (ตัวอย่างการเขียนดูจาก website หน่วยส่งเสริมฯ)

ข้าพเจ้าขอรับรองว่า ข้อความในข้อเสนอโครงการวิจัยฯและสิ่งที่ต้องเสนอพร้อมข้อเสนอโครงการวิจัยฯมีความถูกต้องเป็นจริง หากมีการปรับปรุงแก้ไขเอกสารเกี่ยวกับการวิจัย (revised research document) เป็นต้นว่า การแก้ไขแบบแผนการ ดำเนิน การวิจัย (protocol amendment) การทำให้รายละเอียดเกี่ยวกับผลิตภัณฑ์มีข้อมูลทันสมัย (update

investigator brochure) และการแก้ไขหนังสือยินยอมและเอกสารแนะนำผู้ป่วย (revised consent form/information sheet) ข้าพเจ้าจะแจ้งให้ คณะอนุกรรมการพิจารณาจริยธรรมด้านวิจัยฯ ทราบ

ข้าพเจ้ามี/จัดให้มีกระบวนการลงนามยินยอมอย่างเต็มที่ และ ลงนามใบยินยอมของโครงการวิจัยฯ ตามประเภท โครงการวิจัยฯ หรือ

ข้าพเจ้ามี/จัดให้มีกระบวนการยินยอมด้วยวาจาอย่างเต็มที่ และ ลงนามใบยินยอมของโรงพยาบาล (อยู่โรงพยาบาล หรือ การผ่าตัดที่รุกล้ำ) ตามประเภทโครงการวิจัยฯ

ข้าพเจ้ามี/จัดให้มีกระบวนการ การกำกับ และตรวจสอบตามหลักเกณฑ์การบริบาลผู้ป่วยรวมถึง ติดตาม/ ดูแลด้านความปลอดภัยของผู้ป่วย

ข้าพเจ้าเป็นผู้รายงานความคืบหน้าโครงการวิจัยฯทุก 6 เดือน นับตั้งแต่วันที่ได้รับการรับรองด้านจริยธรรม หรือ ทุกครั้งที่เบิกจ่ายเงินอุดหนุนงวดต่อไป

ข้าพเจ้าเป็นผู้รายงานเหตุการณ์ไม่พึงประสงค์ร้ายแรงของผู้ป่วย ผู้เข้าร่วมโครงการวิจัยฯนี้ ให้หัวหน้าภาควิชา / หน่วยงาน และ ผู้อำนวยการโรงพยาบาลสงขลานครินทร์ทราบ ด้วยแบบรายงานเหตุการณ์ (incidence report) ของโรงพยาบาลฯ พร้อมสำเนาให้ประธานอนุกรรมการพิจารณาจริยธรรมด้านวิจัยฯ ทราบ

ข้าพเจ้ามี/จัดให้มีกระบวนการติดตาม/ชำระความถูกต้องของข้อมูล

ในกรณีเคลื่อนย้ายสิ่งส่งตรวจที่ได้มาจากผู้ป่วยในโครงการวิจัยฯ ทั้งเป็น/ไม่เป็นการบริบาลผู้ป่วย เช่น เลือด สารคัดหลั่ง เนื้อเยื่อและอวัยวะ เพื่อรอตรวจ/ตรวจทางห้องปฏิบัติพิเศษ ณ ต่างประเทศ ข้าพเจ้าต้องให้เสนอขอความเห็น และอนุมัติจากคณะกรรมการฯก่อน ในกรณีมีความคืบหน้าเกี่ยวกับผลการตรวจพิเศษทางห้องปฏิบัติการ ข้าพเจ้าเป็นผู้รายงานให้คณะกรรมการฯทราบ

ข้าพเจ้าขอให้คำมั่นและปฏิบัติตามจรรยาบรรณนักวิจัยฯ ซึ่งประกาศโดยสภาวิจัยแห่งชาติ เมื่อ เสร็จสิ้น โครงการวิจัยฯ ในกรณีรับทุนอุดหนุนจากแหล่งทุนภายนอก ข้าพเจ้าเป็นผู้ทำบทคัดย่อ (abstract) เสนอฝ่ายวิจัย ส่วนกรณี รับทุนอุดหนุนจากกองทุนวิจัย คณะแพทยศาสตร์ ข้าพเจ้าเป็นผู้ทำต้นฉบับพร้อมตีพิมพ์ (manuscript) แบบบทความดั้งเดิม (original article) หรือ บทความสั้น (short article) เสนอฝ่ายวิจัย

ลงชื่อ.....[signature].....

(รศ.พญ.สุภาภรณ์ เต็งไตรสรณ์)

ตำแหน่ง หัวหน้าโครงการ

วันที่ 26 เดือน มิ.ค. พ.ศ. 57

ลงชื่อ.....[signature].....

ลงชื่อ...

(นางสาวศรีระบาย ช่วยจันทร์)

ตำแหน่ง ผู้ร่วมวิจัย

วันที่ 27 เดือน มี.ค. พ.ศ. 57

ลงชื่อ...

(นพ.อรรคพล ตั้งสัตยาธิษฐาน)

ตำแหน่ง ผู้ร่วมวิจัย

วันที่ 27 เดือน มี.ค. พ.ศ. 57

ลงชื่อ...

(ผศ.พญ.เพ็นนี สิงหะ)

ตำแหน่ง ผู้ร่วมวิจัย

วันที่ 27 เดือน มี.ค. พ.ศ. 57

คำอนุมัติจากหัวหน้าภาควิชา/หน่วยงานหรือเทียบเท่า

อนุมัติ

....

ลงชื่อ...

(ผศ.นพ.ธวัช ต้นติสารศาสน์)

ตำแหน่ง หัวหน้าภาควิชาจักษุวิทยา

วันที่ 27 เดือน มี.ค. พ.ศ. 2557
