# Supplementary material for: The reliability of the angle of deviation measurement from the Photo-Hirschberg tests and Krimsky tests
Source: PLoS One. 2021 Dec 1;16(12):e0258744. doi: 10.1371/journal.pone.0258744 (PMC8635364; doi:10.1371/journal.pone.0258744)
Supplement: S6 File — (PDF) [file pone.0258744.s006.pdf]

Clinical Trialproposal form

1.Title: The accuracy of strabismic measurement with Krimsky test

Keywords: Krimsky test, alternate prism cover test, horizontal strabismus, angle of deviation

2.List and duties of the research team **proportion**

Project leader

SUPAPORN TENGTRISORN, MD 40%

Instructor

Department of Ophthalmology, Faculty of Medicine,

Prince of Songkla University

Role: Proposal development

Data collection

Data analysis

Manuscript writing

Co-researcher

SOMPORN BHURACHOKWIWAT 15%

Orthoptist

Department of Ophthalmology, Faculty of Medicine,

Prince of Songkla University

Role: Collecting data

Co-researcher

SRIRABAY CHOUYJAN 15%

Orthoptist

Department of Ophthalmology, Faculty of Medicine,

Prince of Songkla University

Role: Collecting data

Co-researcher

AKKAPOL TUNGSATTHAYATHITHAN, MD 10%

Resident

Department of Ophthalmology, Faculty of Medicine,

Prince of Songkla University

Role: Collecting data

Co-researcher

PENNY SINGHA, MD 10%

Instructor

Department of Ophthalmology, Faculty of Medicine,

Prince of Songkla University

Role: Manuscript writing

Co-researcher

SUPPAKORN NAPHATTHALUNG, MD 10%

Resident

Department of Ophthalmology, Faculty of Medicine,

Prince of Songkla University

Role: Collecting data

3. Background and Rationale:

Strabismus is a common disorder and associated with visual development. In children still have visual development, so strabismus in this age should be early detected and management for improving development. Krimsky test is easy to performed in children because it does not need cooperation but the accuracy depended on experience personnel. The alternate prism cover test is high accurate but it needs to examine in cooperating patients. The investigator is approved for research"The comparison an angle of deviation from photographs with alternate prism cover test in strabismic patient", so we need to do krimsky testin the same patients. In Songklanagarind hospital, we have experience personnel and resources for this research.

4. Objectives:

To compare the angle of deviation measured from Krimsky testing, with that from an alternate prism cover test (APCT) in strabismus patients.

5. Literature review

Joo KSet al. studied in 20 esotropia patients and 20 exotropia patients. The study shows the angle of deviation measured from Krimsky test when distance fixation better than reutein Krimsky test compared to measured from alternate prism cover. That is useful for measuring angle deviation in uncooperative patients Choi RYet al. studied accuracy for measuring angle deviation from Hirschberg test which perform by16 strabismus ophthalmologist. They evaluated angle deviation from photograph of 4 strabismus patients. The

Hirschberg test and Krimsky test are not accurate for measured angle deviation within 10 PD compare to the alternate prism cover.

6. Study design

According to the approved research project"The comparison an angle of deviation from photographs with alternate prism cover test in strabismic patients", the study will measure angle deviation from Krimsky test before alternate prism cover test.

7.Sample selection

According to the approved research project"The comparison an angle of deviation from photographs with alternate prism cover test in strabismic patients."

8. Method:

The patients were measure angle deviation from the Krimsky test then was taken photograph and perform angle deviation with alternate prism cover test. According to the approved research project "The comparison an angle of deviation from photographs with alternate prism cover test in strabismic patients."

9.Patient monitoring:

According to the approved research project "The comparison an angle of deviation from photographs with alternate prism cover test in strabismic patients."

Time for study: 1 April 2013 to 30 December 2019

10.Evaluation of the studied variables

Angle of deviation measured from the Krimsky test and that from the alternate prism cover test

11.Subject:

According to the approved research project "The comparison an angle of deviation from photographs with alternate prism cover test in strabismic patients."

12.Data management

- General data from the approved research project "The comparison an angle of deviation from photographs with alternate prism cover test in strabismic patients."
- An orthoptis measured angle of deviation from the Krimsky test in out patient unit, Songklanagarin hospital.
- To compare the angle of deviation measured from Krimsky testing, with that from an alternate prism cover test (APCT) in strabismus patients.

13.Data analysis

Using Pearson’s Correlationfor correlation analysis between the Krimsky test and the alternate prism cover test

14.Ethical criteria

According to the approved research project “The comparison an angle of deviation from photographs with alternate prism cover test in strabismic patients.”

15.Benefit for the Thai public health system.

To present proper method for measuring angle deviation in rural hospital without experience personnel

16. Faculty of Medicine fund: 8,300 bath

17.References:

1. Joo KS,Koo H, Moon NJ. Measurement of strbismic angle using the distance krimsky test. Korean J Ophthalmol 2013 Aug;27(4):276-81.
2. Choi RY, Kushner BJ. The accracy of experienced strabismologists using the Hirschberg and Krimsky test. Ophthalmology 1998 Jul; 105(7):1301-6.
3. Thompson JT, Guyton DL. Ophthalmic prism. Measurement error and how to minimize them. Ophthalmology 1983;90(3):204-10.

18.Supplement:

- Case record form
- Adverse events andadverse reactions form
- summery for outcome

I certify that The statements in the research proposal and the proposals that must be submitted together with the research proposal are accurate and truthful.

If there is an amendment to the research document such as revised research document, protocol amendment, update investigator brochure and revised consent form/information sheet, I will notify Research Ethics Subcommittee.

I set up a fully informed consent signing process and sign the consent form of the research project according to the type of research project.

I have / set up a supervisory and review process in accordance with the patient care criteria, including Follow / take care of patient safety

I am going to progress report of the research program every 6 months starting from the date of ethical certification or each time the next subsidy disbursement is disbursed.

I am going to report a serious adverse event, participants in this research project to the head of department / department and the director of Songklanagarin hospital with incidence report form together with a copy to the chairman of the ethics review subcommittee on research.

I have established a procedure for tracking / payment of information accuracy in the case of transferring specimens obtained from patients in the trial such as blood, secretion, tissue and organs for examination at a special laboratory abroad. I must seek an opinion and approval from the Board. In the event that there is progress regarding the special laboratory examination results I will report to the Board of Directors for acknowledgment

I hereby make a commitment and abide by the Researcher's Code of Conduct which was announced by the National Research Council. Upon completion of the research project In the case of receiving funding from external sources I am the author of the abstract (abstract) proposing to research department. In the case of receiving funding from the research fund Faculty of Medicine I will write a manuscript, original article or short article, proposed to research department.

sign..

SUPAPORN TENGTRISORN)

Project leader

Date…………………….........

sign..

(SOMPORN BHURACHOKWIWAT)

Co-researcher

Date…………………….........

sign..

(SRIRABAY CHOUYJAN)

Co-researcher

Date…………………….........

sign...

(AKKAPOL TUNGSATTHAYATHITHAN)

Co-researcher

Date…………………….........

sign...

(PENNY SINGHA)

Co-researcher

Date…………………….........

sign...

(SUPPAKORN NAPHATTHALUNG)

Co-researcher

Date…………………….........

Approval from the head of the department

....

....

sign...

(Assist. Prof. Tawat Tantisarasart)

Head of department

Date…………………….........
