# Supplementary material for: The reliability of the angle of deviation measurement from the Photo-Hirschberg tests and Krimsky tests
Source: PLoS One. 2021 Dec 1;16(12):e0258744. doi: 10.1371/journal.pone.0258744 (PMC8635364; doi:10.1371/journal.pone.0258744)
Supplement: S8 File — (PDF) [file pone.0258744.s008.pdf]

no	gender	age	age_gr	age_gr2	prefer
1	2	20	2	2	1
2	1	38	2	2	2
3	1	27	2	2	1
4	1	6	1	1	2
5	1	48	2	2	1
7	2	5	1	1	2
8	2	5	1	1	2
9	2	31	2	2	2
11	2	14	1	2	1
12	2	9	1	1	2
13	1	6	1	1	1
14	1	10	1	1	2
15	1	6	1	1	2
16	1	71	2	2	2
17	1	7	1	1	1
18	2	8	1	1	2
19	2	6	1	1	1
20	1	10	1	1	1
21	2	8	1	1	1
23	1	6	1	1	2
24	1	5	1	1	2
25	2	6	1	1	1
26	1	6	1	1	1
27	1	16	1	2	2
29	2	7	1	1	1
31	2	6	1	1	1
32	2	7	1	1	1
33	1	45	2	2	1

actual	RE1m	LE1m	CD1m	AB1m	mm1m	ESTmm1m	RE4m	LE4m	CD4m	AB4m
1	5.04	7.81	12.85	10.75	2.10	-2.10	5.12	7.85	12.97	10.28
1	8.32	5.04	13.36	10.27	3.09	-3.09	7.21	6.28	13.49	10.86
2	5.98	3.45	9.43	11.79	2.36	2.36	5.34	3.15	8.49	11.98
1	7.72	5.60	13.32	10.67	2.65	-2.65	8.04	5.43	13.47	10.87
2	5.22	2.88	8.10	10.46	2.36	2.36	5.91	1.57	7.48	11.25
1	9.50	5.58	15.08	11.21	4.07	-4.07	8.50	6.11	14.61	10.71
1	7.22	4.45	11.67	10.14	1.53	-1.53	7.90	4.65	12.55	10.47
2	3.89	6.00	9.89	12.40	2.51	2.51	3.65	6.35	10.00	12.90
2	4.24	5.15	9.39	10.74	1.35	1.35	3.82	5.14	8.96	10.79
2	3.25	5.62	8.87	11.96	3.09	3.09	3.32	5.63	8.95	12.12
1	6.04	7.62	13.66	12.46	1.20	-1.20	6.22	7.08	13.30	12.27
1	7.02	5.33	12.35	10.33	2.02	-2.02	7.05	5.52	12.57	10.39
2	5.66	4.54	10.20	11.36	1.16	1.16	4.82	4.92	9.74	11.17
2	1.52	5.62	7.14	11.05	3.91	3.91	1.05	5.22	6.27	11.58
1	5.55	7.21	12.76	10.51	2.25	-2.25	6.82	4.88	11.70	9.97
1	8.15	5.62	13.77	10.20	3.57	-3.57	7.93	5.18	13.11	10.39
1	5.15	6.72	11.87	10.40	1.47	-1.47	7.06	5.33	12.39	10.09
1	6.63	6.42	13.05	10.61	2.64	-2.64	6.02	6.53	12.55	10.55
1	7.04	7.45	14.49	13.10	1.39	-1.39	5.82	7.82	13.64	11.57
1	8.67	5.44	14.11	11.11	3.00	-3.00	7.45	5.95	13.40	10.69
2	3.12	4.90	8.02	10.38	2.36	2.36	3.78	4.50	8.28	10.26
1	6.95	5.55	12.50	10.72	1.78	-1.78	5.24	7.62	12.86	10.76
1	5.84	8.62	14.46	11.05	3.41	-3.41	5.24	8.62	13.86	10.50
1	7.00	5.52	12.52	11.17	1.35	-1.35	7.55	5.89	13.44	10.38
1	4.65	8.37	13.02	9.83	3.19	-3.19	5.24	8.42	13.66	10.87
2	6.25	3.54	9.79	12.02	2.23	2.23	6.45	3.54	9.99	12.62
2	5.20	5.40	10.60	12.20	1.60	1.60	7.25	3.15	10.40	11.02
2	5.25	2.54	2.79	10.95	3.16	3.16	4.62	3.25	7.87	11.70

mm4m	ESTmm4m	mphoto1m	mestpd1m	photo1m	estpd1m	gold1m	ESTgold1m
2.69	-2.69	40.05	-40.05	44.75	-44.75	50.00	-50.00
2.63	-2.63	58.93	-58.93	65.85	-65.85	40.00	-40.00
3.49	3.49	45.01	45.01	40.76	40.76	50.00	50.00
2.60	-2.60	50.54	-50.54	56.47	-56.47	60.00	-60.00
3.77	3.77	45.00	45.00	40.76	40.76	80.00	80.00
4.44	-4.44	77.61	-77.61	86.73	-86.73	50.00	-50.00
2.08	-2.08	29.18	-29.18	32.60	-32.60	40.00	-40.00
2.90	2.90	47.87	47.87	43.35	43.35	47.00	47.00
1.83	1.83	25.74	25.74	23.31	23.31	60.00	60.00
3.17	3.17	58.93	58.93	53.36	53.36	45.00	45.00
1.03	-1.03	22.88	-22.88	25.57	-25.57	35.00	-35.00
2.18	-2.18	38.52	-38.52	43.05	-43.05	35.00	-35.00
1.43	1.43	22.12	22.12	20.03	20.03	25.00	25.00
5.31	5.31	74.56	74.56	67.53	67.53	80.00	80.00
1.73	-1.73	42.91	-42.91	47.95	-47.95	45.00	-45.00
2.72	-2.72	68.08	-68.08	76.08	-76.08	60.00	-60.00
2.30	-2.30	28.03	-28.03	31.33	-31.33	65.00	-65.00
2.00	-2.00	50.34	-50.34	56.26	-56.26	40.00	-40.00
2.07	-2.07	26.51	-26.51	29.62	-29.62	37.00	-37.00
2.71	-2.71	57.21	-57.21	63.93	-63.93	60.00	-60.00
2.98	2.98	45.01	45.01	40.76	40.76	50.00	50.00
2.10	-2.10	33.94	-33.94	37.93	-37.93	45.00	-45.00
3.36	-3.36	65.03	-65.03	72.57	-72.57	75.00	-75.00
3.06	-3.06	25.74	-25.74	28.77	-28.77	45.00	-45.00
2.79	-2.79	60.83	-60.83	67.98	-67.98	45.00	-45.00
2.63	2.63	42.53	42.53	38.51	38.51	35.00	35.00
0.62	0.62	30.51	30.51	27.63	27.63	25.00	25.00
3.83	3.83	60.26	60.26	54.57	54.57	90.00	90.00

mdiff1m	diff1m	diff1mgr	mphoto4m	mestpd4m	photo4m	estpd4m	gold4m
-9.95	-5.25	2	47.37	-47.37	54.23	-54.23	50.00
18.93	25.85	4	46.31	-46.31	53.02	-53.02	40.00
-4.99	-9.24	2	61.46	61.46	54.55	54.55	50.00
-9.46	-3.53	2	45.79	-45.79	52.42	-52.42	55.00
-35.00	-39.24	1	66.39	66.39	58.93	58.93	75.00
27.61	36.73	4	78.19	-78.19	89.51	-89.51	50.00
-10.82	-7.40	2	36.63	-36.63	41.93	-41.93	40.00
0.87	-3.65	2	51.07	51.07	45.33	45.33	47.00
-34.26	-36.69	1	32.23	32.23	28.60	28.60	60.00
13.93	8.36	3	55.82	55.82	49.55	49.55	45.00
-12.12	-9.43	2	18.14	-18.14	20.76	-20.76	35.00
3.52	8.05	3	38.39	-38.39	43.95	-43.95	37.00
-2.88	-4.97	2	25.18	25.18	22.35	22.35	25.00
-5.44	-12.47	1	93.51	93.51	83.00	83.00	80.00
-2.09	2.95	3	30.47	-30.47	34.88	-34.88	47.00
8.08	16.08	4	47.90	-47.90	54.84	-54.84	60.00
-36.97	-33.67	1	40.50	-40.50	46.37	-46.37	60.00
10.34	16.26	4	35.22	-35.22	40.32	-40.32	42.00
-10.49	-7.38	2	36.45	-36.45	41.73	-41.73	35.00
-2.79	3.93	3	47.72	-47.72	54.63	-54.63	60.00
-4.99	-9.24	2	34.87	34.87	30.95	30.95	50.00
-11.06	-7.07	2	36.98	-36.98	42.34	-42.34	45.00
-9.97	-2.43	2	64.08	-64.08	67.74	-67.74	75.00
-19.26	-16.23	1	53.89	-53.89	61.69	-61.69	45.00
15.83	22.98	4	49.13	-49.13	56.25	-56.25	45.00
7.53	3.51	3	46.31	46.31	41.11	41.11	35.00
5.51	2.63	3	10.92	10.92	9.96	9.96	25.00
-29.74	-35.43	1	67.45	67.45	59.86	59.86	90.00

ESTgold4m	mdiff4m	diff4m	diff4mgr	cdifer1m	cdifle1m	cdifre4m	cdifle4m
-50.00	-2.63	4.23	3	-1.56	-1.59	-1.74	-1.53
-40.00	6.31	13.02	4	-1.72	-1.22	-0.74	-2.59
50.00	11.46	4.55	3	2.88	2.57	1.78	1.47
-55.00	-9.21	-2.58	2	-1.95	-0.60	-1.77	-1.47
75.00	-8.61	-16.07	1	3.34	2.76	3.24	4.94
-50.00	28.19	39.51	4	-1.52	-0.69	-3.32	-2.89
-40.00	-3.37	1.93	3	-1.64	-2.01	-1.47	-1.62
47.00	4.07	-1.67	2	2.98	2.69	3.47	2.46
60.00	-27.77	-31.40	1	1.19	1.71	-0.05	0.39
45.00	10.82	4.55	3	2.57	2.77	2.85	2.64
-35.00	-16.86	-14.24	1	-1.27	-1.78	-0.79	-1.29
-37.00	1.39	6.95	3	-0.97	-0.57	-2.02	-2.02
25.00	0.18	-2.65	2	0.94	0.74	0.97	1.66
80.00	13.51	3.00	3				
-47.00	-16.53	-12.12	1	-1.63	-1.51	-1.52	-1.27
-60.00	-12.10	-5.16	2	-0.63	1.79	1.73	-2.16
-60.00	-19.50	-13.63	1	-1.36	-1.53	-2.33	-2.07
-42.00	-6.78	-1.68	2	-0.60	-1.19	-0.73	-0.73
-35.00	1.45	6.73	3	-0.81	-0.94	-0.94	-0.79
-60.00	-12.28	-5.37	2	-2.07	-0.80	-1.89	-2.12
50.00	-15.13	-19.05	1	1.89	1.89	1.61	1.76
-45.00	-8.02	-2.66	2	-1.57	-1.71	-0.40	-0.40
-75.00	-10.92	-7.26	2	-2.11	-7.15	-1.88	-1.80
-45.00	8.89	16.69	4	-1.70	-1.82	-0.38	-0.22
-45.00	4.13	11.25	4	-0.29	1.11	-6.83	-4.66
35.00	11.31	6.11	3				
25.00	-14.08	-15.04	1				
90.00	-22.55	-30.14	1				

cmpho1m	cpho1m	cmpho4m	cpho4m	a1	b1	a4	b4	cclrn
-29.67	-33.16	-30.63	-35.06	20.33	16.84	19.37	14.94	0.5360824
-23.32	-26.06	-45.67	-52.28	16.68	13.94	5.67	12.28	0.6071429
55.00	49.80	31.35	27.82	5.00	0.20	18.65	22.18	0.3830228
-11.38	-12.72	-25.90	-29.65	48.62	47.28	29.10	25.35	0.5876011
63.72	57.70	57.06	50.64	16.28	22.30	17.94	24.36	0.3754941
-13.07	-14.61	-50.81	-58.16	36.93	35.39	0.81	8.16	0.5995145
-38.43	-42.94	-28.50	-32.62	1.57	2.94	11.50	7.38	0.5211267
51.21	46.21	43.24	38.38	4.21	0.79	3.76	8.62	0.3790323
22.67	20.53	-0.81	-0.92	37.33	39.47	60.81	60.92	0.4043321
52.89	47.90	46.45	41.23	7.89	2.90	1.45	3.77	0.3854545
-24.22	-27.07	-13.90	-15.91	10.78	7.93	21.10	19.09	0.5444444
-10.89	-12.17	-35.62	-40.78	24.11	22.83	1.38	3.78	0.5204918
14.03	12.70	29.16	25.88	10.97	12.30	4.16	0.88	0.4051724
								0.2746269
-31.02	-34.66	-26.70	-30.56	13.98	10.34	20.30	16.44	0.543554
34.18	30.96	-37.97	-43.47	94.18	90.96	22.03	16.53	0.6053269
-25.89	-28.93	-40.99	-46.93	39.11	36.07	19.01	13.07	0.5136778
-11.53	-12.88	-12.93	-14.80	28.47	27.12	29.07	27.20	0.5017668
-15.46	-17.27	-16.57	-18.97	21.54	19.73	18.43	16.03	0.5152543
-15.24	-17.03	-37.33	-42.73	44.76	42.97	22.67	17.27	0.5888889
36.06	32.65	30.93	27.45	13.94	17.35	19.07	22.55	0.3754153
-29.90	-33.42	-7.02	-8.04	15.10	11.58	37.98	36.96	0.5373832
-40.21	-44.93	-33.02	-37.80	34.79	30.07	41.98	37.20	0.5864662
-34.70	-38.78	-3.79	-4.34	10.30	6.22	41.21	40.66	0.5582525
-5.53	-6.18	-120.27	-137.69	39.47	38.82	75.27	92.69	0.584022
								0.3773585
								0.414966
								0.2471483

cclrd	cclrngr	cclrdgr	ndgr	kim1m	estkim1m
0.5366973	2	2	22		
0.5859031	2	2	22	50	-50
0.3804627	2	2	22	50	50
0.5741758	2	2	22	55	-55
0.3211382	2	2	22	50	50
0.5902062	2	2	22	65	-65
0.5229682	2	2	22		
0.3703704	2	2	22	55	55
0.3843284	2	2	22	50	50
0.380597	2	2	22	50	50
0.5375375	2	2	22	35	-35
0.5362903	2	2	22	30	-30
0.4224138	2	1	21	30	30
0.2895522	2	2	22	70	70
0.5069444	2	2	22	40	-40
0.5953488	2	2	22	60	-60
0.5166163	2	2	22	40	-40
0.5036497	2	2	22	40	-40
0.5273973	2	2	22	30	-30
0.5696721	2	2	22	60	-60
0.3661017	2	2	22	45	45
0.5555556	2	2	22	40	-40
0.5766423	2	2	22	65	-65
0.5565611	2	2	22	40	-40
0.5821727	2	2	22	50	-50
0.3766234	2	2	22	50	50
0.4	2	2	22		
0.2592593	2	2	22	85	85

kim4m	estkim4m	diffkim1m	diffkim1mgr	diffkim4m	diffkim4mgr
50	-50	10.00	3	10.00	3
50	50	0.00	0	0.00	0
50	-50	-5.00	2	-5.00	2
50	50	-30.00	1	-25.00	1
65	-65	15.00	4	15.00	4
55	55	8.00	3	8.00	3
50	50	-10.00	2	-10.00	2
55	55	5.00	3	10.00	3
35	-35	0.00	0	0.00	0
30	-30	-5.00	2	-7.00	2
35	35	5.00	3	10.00	3
65	65	-10.00	2	-15.00	1
35	-35	-5.00	2	-12.00	1
60	-60	0.00	0	0.00	0
35	-35	-25.00	1	-25.00	1
35	-35	0.00	0	-7.00	2
25	-25	-7.00	2	-10.00	2
60	-60	0.00	0	0.00	0
45	45	-5.00	2	-5.00	2
45	-45	-5.00	2	0.00	0
65	-65	-10.00	2	-10.00	2
40	-40	-5.00	2	-5.00	2
50	-50	5.00	3	5.00	3
50	50	15.00	4	15.00	4
85	85	-5.00	2	-5.00	2
