# Supplementary material for: The reliability of the angle of deviation measurement from the Photo-Hirschberg tests and Krimsky tests
Source: PLoS One. 2021 Dec 1;16(12):e0258744. doi: 10.1371/journal.pone.0258744 (PMC8635364; doi:10.1371/journal.pone.0258744)
Supplement: S1 Table — (DOCX) [file pone.0258744.s009.docx]

**Table 1** Summary results of correlation between angle of deviation from the Photo-Hirschberg test with the APCT.

| **Variable** | **At N, n(%)** | | | **At D, n(%)** | | |
| --- | --- | --- | --- | --- | --- | --- |
|  | **Total** | **ET** | **XT** | **Total** | **ET** | **XT** |
| Absolute difference within acceptable range | 17 (60.7) | 10 (58.8) | 7 (63.6) | 16 (57.1) | 10 (58.8) | 6 (54.5) |
| Area b | 6 (21.4) | 3 (17.6) | 3 (27.3) | 8 (28.6) | 4 (23.5) | 4 (36.4) |
| Area c | 11 (39.3) | 7 (41.2) | 4 (36.4) | 8 (28.6) | 6 (35.3) | 2 (18.2) |
| Absolute difference within unacceptable range | 11 (39.3) | 7 (41.2) | 4 (36.4) | 12 (42.9) | 7 (41.2) | 5 (45.5) |
| Area a | 5 (17.9) | 5 (29.4) | 0 (0) | 4 (14.3) | 4 (25.5) | 0 (0) |
| Area d | 6 (21.4) | 2 (11.8) | 4 (36.4) | 8 (28.6) | 3 (17.6) | 5 (45.5) |

APCT = alternate prism cover test, ET = Esotropia, XT = Exotropia, PD = prism diopter, = n number of subjects**,** N = near fixation, D = distance fixation
