# Supplementary material for: The reliability of the angle of deviation measurement from the Photo-Hirschberg tests and Krimsky tests
Source: PLoS One. 2021 Dec 1;16(12):e0258744. doi: 10.1371/journal.pone.0258744 (PMC8635364; doi:10.1371/journal.pone.0258744)
Supplement: S2 Table — (DOCX) [file pone.0258744.s010.docx]

**Table 2** Summary results of correlation between angle of deviation from the Krimsky with the APCT.

| **Variable** | **At N2, n(%)** | | | **At D2, n(%)** | | |
| --- | --- | --- | --- | --- | --- | --- |
|  | **Total** | **ET** | **XT** | **Total** | **ET** | **XT** |
| Equality | 5 (20) | 4 (26.7) | 1 (10) | 5 (20) | 4 (26.7) | 1 (10) |
| Absolute difference within acceptable range | 16 (64) | 9 (60) | 7 (70) | 14 (56) | 8 (53.3) | 6 (60) |
| Area b | 5 (20) | 2 (13.3) | 3 (30) | 5 (20) | 2 (13.3) | 3 (30) |
| Area c | 11 (44) | 7 (46.7) | 4 (40) | 9 (36) | 6 (40) | 3 (30) |
| Absolute difference within unacceptable range | 4 (16) | 2 (13.3) | 2 (20) | 6 (24) | 3 (20) | 3 (30) |
| Area a | 2 (8) | 1 (6.7) | 1 (10) | 2 (8) | 1 (6.7) | 1 (10) |
| Area d | 2 (8) | 1 (6.7) | 1 (10) | 4 (16) | 2 (13.3) | 2 (20) |

APCT = alternate prism cover test, ET = Esotropia, XT = Exotropia, PD = prism diopter, n = number of subjects, N2= near fixation at 30 cm, D2=distance fixation at 6 m
