# Supplementary material for: The reliability of the angle of deviation measurement from the Photo-Hirschberg tests and Krimsky tests
Source: PLoS One. 2021 Dec 1;16(12):e0258744. doi: 10.1371/journal.pone.0258744 (PMC8635364; doi:10.1371/journal.pone.0258744)
Supplement: Attachment — Submitted filename: response to reviewers.docx [file pone.0258744.s011.docx]

PLOS ONE Decision: Revision required [PONE-D-21-11420] - [EMID:b9b26f14883e9bd3]

em.pone.0.746eb1.f93d9601@editorialmanager.com

 on behalf of

PLOS ONE <em@editorialmanager.com>

Mon 7/5, 7:46 PM

SUPAPORN TENGIRISORN

Inbox

Flag for follow up. Start by Tuesday, July 06, 2021. Due by Tuesday, July 06, 2021.

Action Items

PONE-D-21-11420
The reliability of the angle of deviation measurement from Photo-Hirschberg tests and Krimsky tests
PLOS ONE

Dear Dr. tengtrisorn,

Thank you for submitting your manuscript to PLOS ONE. After careful consideration, we feel that it has merit but does not fully meet PLOS ONE’s publication criteria as it currently stands. Therefore, we invite you to submit a revised version of the manuscript that addresses the points raised during the review process.

The idea of the manuscript is interesting However, the manuscript needs some justification for the exclusion of  some patients, and explanation of how patients were intermittent exotropia were photpgraphed..

ANS: We need to set the research protocol in real world, so intermittent exotropia was not in the exclusion criteria. After we included all subjects and analyzed, we try to explain for someone who needs to use the data in clinical practice. After your recommend we reanalyzed after excluded intermittent strabismus cases.

Please submit your revised manuscript by Aug 19 2021 11:59PM. If you will need more time than this to complete your revisions, please reply to this message or contact the journal office at plosone@plos.org. When you're ready to submit your revision, log on to https://www.editorialmanager.com/pone/ and select the 'Submissions Needing Revision' folder to locate your manuscript file.

Please include the following items when submitting your revised manuscript:

- A rebuttal letter that responds to each point raised by the academic editor and reviewer(s). You should upload this letter as a separate file labeled 'Response to Reviewers'.
- A marked-up copy of your manuscript that highlights changes made to the original version. You should upload this as a separate file labeled 'Revised Manuscript with Track Changes'.
- An unmarked version of your revised paper without tracked changes. You should upload this as a separate file labeled 'Manuscript'.

If you would like to make changes to your financial disclosure, please include your updated statement in your cover letter. Guidelines for resubmitting your figure files are available below the reviewer comments at the end of this letter.

ANS: I changed my financial disclosure and include in cover letter.

If applicable, we recommend that you deposit your laboratory protocols in protocols.io to enhance the reproducibility of your results. Protocols.io assigns your protocol its own identifier (DOI) so that it can be cited independently in the future. For instructions see: <http://journals.plos.org/plosone/s/submission-guidelines#loc-laboratory-protocols>. Additionally, PLOS ONE offers an option for publishing peer-reviewed Lab Protocol articles, which describe protocols hosted on protocols.io. Read more information on sharing protocols at <https://plos.org/protocols?utm_medium=editorial-email&utm_source=authorletters&utm_campaign=protocols>.

We look forward to receiving your revised manuscript.

Kind regards,

Ahmed Awadein, MD, Ph.D, FRCS
Academic Editor
PLOS ONE

Journal Requirements:

When submitting your revision, we need you to address these additional requirements.

1. Please ensure that your manuscript meets PLOS ONE's style requirements, including those for file naming. The PLOS ONE style templates can be found at

https://journals.plos.org/plosone/s/file?id=wjVg/PLOSOne_formatting_sample_main_body.pdf and

[https://journals.plos.org/plosone/s/file?id=ba62/PLOSOne_formatting_sample_title_authors_affiliations.pdf](about:blank)

2. Thank you for submitting your clinical trial to PLOS ONE and for providing the name of the registry and the registration number. The information in the registry entry suggests that your trial *TCTR20141201001* was registered after patient recruitment began. PLOS ONE strongly encourages authors to register all trials before recruiting the first participant in a study.

As per the journal’s editorial policy, please include in the Methods section of your paper:
1) your reasons for your delay in registering this study (after enrolment of participants started);

ANS: The ethics committee was approved the research protocol then we made registration of the protocol to TCTR before we started to recruit subjects. However, we did not check for completion of registration.

2) confirmation that all related trials are registered by stating: “The authors confirm that all ongoing and related trials for this drug/intervention are registered”.

 ANS: We confirmed that all related trials are registered by stating: “The authors confirm that all ongoing and related trials for this drug/intervention are registered”.

3. Please include your tables as part of your main manuscript and remove the individual files. Please note that supplementary tables should remain uploaded as separate "supporting information" files.

ANS: Changed

4. Thank you for stating the following in the Acknowledgments Section of your manuscript:

"This research was partially supported by a grant from the Faculty of Medicine, Prince of Songkla University."-

ANS: I rewrite it from the manuscript.

We note that you have provided funding information that is not currently declared in your Funding Statement. However, funding information should not appear in the Acknowledgments section or other areas of your manuscript. We will only publish funding information present in the Funding Statement section of the online submission form.

Please remove any funding-related text from the manuscript and let us know how you would like to update your Funding Statement. Currently, your Funding Statement reads as follows:

"No"

Please provide an amended statement that declares *all* the funding or sources of support (whether external or internal to your organization) received during this study, as detailed online in our guide for authors at [http://journals.plos.org/plosone/s/submit-now](about:blank).  Please also include the statement “There was no additional external funding received for this study.” in your updated Funding Statement.

Please include your amended statements within your cover letter; we will change the online submission form on your behalf.

ANS: I include the statement “There was no additional external funding received for this study.” in my updated Funding Statement and put it in my cover letter

5. Thank you for stating the following in your Competing Interests section:

"No"

Please complete your Competing Interests on the online submission form to state any Competing Interests. If you have no competing interests, please state "The authors have declared that no competing interests exist.", as detailed online in our guide for authors at [http://journals.plos.org/plosone/s/submit-now](about:blank)

 This information should be included in your cover letter; we will change the online submission form on your behalf.

ANS: On behalf of all authors the corresponding author declares that no competing interests exist and put it in cover letter.

Please know it is PLOS ONE policy for corresponding authors to declare, on behalf of all authors, all potential competing interests for the purposes of transparency. PLOS defines a competing interest as anything that interferes with, or could reasonably be perceived as interfering with, the full and objective presentation, peer review, editorial decision-making, or publication of research or non-research articles submitted to one of the journals. Competing interests can be financial or non-financial, professional, or personal. Competing interests can arise in relationship to an organization or another person. Please follow this link to our website for more details on competing interests: [http://journals.plos.org/plosone/s/competing-interests](about:blank)

6. Please include captions for your Supporting Information files at the end of your manuscript, and update any in-text citations to match accordingly. Please see our Supporting Information guidelines for more information: [http://journals.plos.org/plosone/s/supporting-information](about:blank).

ANS: no other supporting information files

7. We note that Figure 2 includes an image of a patient / participant in the study.

As per the PLOS ONE policy ([http://journals.plos.org/plosone/s/submission-guidelines#loc-human-subjects-research](about:blank)) on papers that include identifying, or potentially identifying, information, the individual(s) or parent(s)/guardian(s) must be informed of the terms of the PLOS open-access (CC-BY) license and provide specific permission for publication of these details under the terms of this license. Please download the [Consent Form for Publication in a PLOS Journal](about:blank) ([http://journals.plos.org/plosone/s/file?id=8ce6/plos-consent-form-english.pdf](about:blank)). The signed consent form should not be submitted with the manuscript, but should be securely filed in the individual's case notes. Please amend the methods section and ethics statement of the manuscript to explicitly state that the patient/participant has provided consent for publication: “The individual in this manuscript has given written informed consent (as outlined in PLOS consent form) to publish these case details”.

If you are unable to obtain consent from the subject of the photograph, you will need to remove the figure and any other textual identifying information or case descriptions for this individual.

ANS: I changed the Figure 2 and no longer a photograph.

[Note: HTML markup is below. Please do not edit.]

Reviewers' comments:

Reviewer's Responses to Questions

**Comments to the Author**

1. Is the manuscript technically sound, and do the data support the conclusions?

The manuscript must describe a technically sound piece of scientific research with data that supports the conclusions. Experiments must have been conducted rigorously, with appropriate controls, replication, and sample sizes. The conclusions must be drawn appropriately based on the data presented.

Reviewer #1: Yes

Reviewer #2: Partly

2. Has the statistical analysis been performed appropriately and rigorously?

Reviewer #1: Yes

Reviewer #2: No

ANS: We reanalyze the manuscript.

3. Have the authors made all data underlying the findings in their manuscript fully available?

The [PLOS Data policy](http://www.plosone.org/static/policies.action#sharing) requires authors to make all data underlying the findings described in their manuscript fully available without restriction, with rare exception (please refer to the Data Availability Statement in the manuscript PDF file). The data should be provided as part of the manuscript or its supporting information, or deposited to a public repository. For example, in addition to summary statistics, the data points behind means, medians and variance measures should be available. If there are restrictions on publicly sharing data—e.g. participant privacy or use of data from a third party—those must be specified.

Reviewer #1: Yes

Reviewer #2: Yes

4. Is the manuscript presented in an intelligible fashion and written in standard English?

PLOS ONE does not copyedit accepted manuscripts, so the language in submitted articles must be clear, correct, and unambiguous. Any typographical or grammatical errors should be corrected at revision, so please note any specific errors here.

Reviewer #1: Yes

Reviewer #2: Yes

5. Review Comments to the Author

Please use the space provided to explain your answers to the questions above. You may also include additional comments for the author, including concerns about dual publication, research ethics, or publication ethics. (Please upload your review as an attachment if it exceeds 20,000 characters)

Reviewer #1: This study addresses an important issue and worked on a technique that may be useful in areas with no strabismus specialist. Although the idea is good yet the study is limited by the small number of cases and major exclusion criteria. The manuscript needs a lot of work to be suitable for publication. Below are the details.

Line 58: “Many studies have tried… “ What are those studies? No references mentioned.

Ans: Changed

Lines 55 to 82: This part should be moved to the discussion. The details of previous investigators’ approach can be included briefly in introduction only if needed to explain the purpose of the study. This section should be reduced in introduction and moved mostly to discussion.

ANS: Changed
Line 89: “Four subjects in the XT group were intermittent XT”: The authors described the diagnosis of four subjects out of the total cohort only. It is unclear why this diagnosis was singled out specifically here. Why not mention the breakdown of the diagnosis of all the test subjects? –

ANS: We reanalyze and rewrite the manuscript after excluded intermittent strabismus cases.

Line 87-94: Inclusion criteria were mentioned, then diagnosis of 4 subjects, then the consent, then back to exclusion criteria. This order is not usual. I recommend keeping the exclusion criteria right after the inclusion criteria.

ANS: changed

Lines 91-94: Why was accommodative ET excluded? Why were cases with amblyopia or pervious surgery excluded? These subgroups are very common diagnoses, especially in the difficult cases that will need to be sent to an expert for opinion. By such exclusions authors are suggesting that the test is no useful on these subgroups and hence not useful for a big chunk of the cases in the strabismus clinic. Cause of such exclusions should be discussed.

ANS: Accmmodation ET showed variation of angle and depend on accommodation power and glasses correction so it may confound the interpretation.

Amblyopia case will have some problem for fixation when we measure angle of deviation
from alternate prism cover test (APCT).

Previous surgery may disturb the action of the eye muscle.

Lines 94-96: Revise sentence structure and avoid repetition (subjects received eye examination)

ANS: Changed

Line 96: I was expecting detailed description of how to Photo-Hirschberg test was done. This is mentioned later lines 108 onwards. I think this should be moved to be in the right place. It is confusing to have to go back and forth in the manuscript. As such it lacks the smoothness that readers expect.
ANS: Changed

Lines 99-100: “Time span..” It is not clear what the authors mean by time span. Time span between what and what? And why is that? This “time span” needs detailed description.

ANS: Changed

Lines 101 -106: The consent is repeated again here. It is mentioned earlier in line 90! I would recommend starting the methodology by stating the approval by the IRB and the consent and then proceeding with the rest of the methodology. Line 105: “and showed.. “ till the end should be deleted. At this point of reading the study didn’t show anything yet. We are still in the methods.

ANS: Changed

Line 117: “limbuson”: seems a spelling mistake

ANS: Changed

Line 118: “When the right eye is fixated”: I think the authors mean when the right eye “is fixing”. Please correct this throughout the text.

ANS: Changed


Line 108: How did the photographer deal with cases of intermittent exotropia to show the deviation?
ANS: We reanalyzed and rewrite the manuscript after excluded intermittent strabismus cases. We suggest to improve the method with using an infrared filter.

Lines 170-187: All this data should be in detailed in the table and deleted from the text. It is very confusing to read or compare these number in paragraph format. A table only without text is enough to show results like this.

ANS: Changed

Lines 189-198: the first paragraph of the discussion is almost all a repetition from the introduction.
ANS: Changed

Lines 213-215: The angles of the cases should be in the results section. The analysis as to why this might have happened goes into the discussion section.

ANS: Changed

Lines 217: Do authors want to implicate that their method is not useful in intermittent exotropia cases? Need clarifications as to what is the authors conclusions about the use in intermittent exotropia cases.

ANS: Changed


Line 234: Conclusion should be in relation to the title and objective of the study. The study is testing “the reliability of Photo-Hirschberg and Krimsky”. The objective as per the abstract “to compare photo-H, Krimsky and APCT”. therefore this should be mentioned in the conclusion. This sentence “ Photo-Hirschberg test could be used to examine and monitor” is not informative. Any test could be used to measure and monitor! Same for saying “Krimsky can be used in uncooperative patients”: this is again basic knowledge and not the conclusion of this study.

ANS: We changed conclusion.

Reviewer #2: The assessment of measurement reliability should not be done by Pearson's correlation but use the Intrasclass Correlation (ICC). Please refer to the recent comments on measurement reliability and its importance for individual differences research (https://www.nature.com/articles/s41562-019-0655-x). In https://www.sciencedirect.com/science/article/pii/S2095927318305784, the anatomy of ICC is introduced. Please update all the reliability analyses in terms of the basic requirement by the two references abovementioned.

ANS: the reliability done by number of subjected within acceptable ranges ( within +/-10 PD of the APCT values). I study ICC from previous references but, I am quite sure that its fit for our paper.

6. PLOS authors have the option to publish the peer review history of their article ([what does this mean?](https://journals.plos.org/plosone/s/editorial-and-peer-review-process#loc-peer-review-history)). If published, this will include your full peer review and any attached files.


If you choose “no”, your identity will remain anonymous but your review may still be made public.


**Do you want your identity to be public for this peer review?** For information about this choice, including consent withdrawal, please see our [Privacy Policy](https://www.plos.org/privacy-policy).

Reviewer #1: **Yes:** Amr ElKamshoushy

Reviewer #2: No

[NOTE: If reviewer comments were submitted as an attachment file, they will be attached to this email and accessible via the submission site. Please log into your account, locate the manuscript record, and check for the action link "View Attachments". If this link does not appear, there are no attachment files.]

While revising your submission, please upload your figure files to the Preflight Analysis and Conversion Engine (PACE) digital diagnostic tool, <https://pacev2.apexcovantage.com/>. PACE helps ensure that figures meet PLOS requirements. To use PACE, you must first register as a user. Registration is free. Then, login and navigate to the UPLOAD tab, where you will find detailed instructions on how to use the tool. If you encounter any issues or have any questions when using PACE, please email PLOS at [figures@plos.org](mailto:figures@plos.org). Please note that Supporting Information files do not need this step.
